# Gene expression in developing watermelon fruit

**DOI:** 10.1186/1471-2164-9-275

**Published:** 2008-06-05

**Authors:** W Patrick Wechter, Amnon Levi, Karen R Harris, Angela R Davis, Zhangjun Fei, Nurit Katzir, James J Giovannoni, Ayelet Salman-Minkov, Alvaro Hernandez, Jyothi Thimmapuram, Yaakov Tadmor, Vitaly Portnoy, Tova Trebitsh

**Affiliations:** 1 USDA, ARS, U.S. Vegetable Lab, 2700 Savannah Highway, Charleston, SC, USA; 2 USDA, ARS, South Central Agricultural Research Laboratory, P.O. Box 159 Hwy 3 West, Lane, OK, USA; 3 USDA, ARS, Robert Holly Center and Boyce Thompson Institute for Plant Research, Tower Road, Ithaca, NY, USA; 4 Agricultural Research Organization, P.O. Box 1021, Ramat Yishay 30095, Israel; 5 Department of Life Sciences, Ben-Gurion University of the Negev, Beer-Sheva, Israel; 6 University of Illinois at Urbana-Champaign, 1201 W. Gregory Drive, Urbana, IL 61801, USA

## Abstract

**Background:**

Cultivated watermelon form large fruits that are highly variable in size, shape, color, and content, yet have extremely narrow genetic diversity. Whereas a plethora of genes involved in cell wall metabolism, ethylene biosynthesis, fruit softening, and secondary metabolism during fruit development and ripening have been identified in other plant species, little is known of the genes involved in these processes in watermelon. A microarray and quantitative Real-Time PCR-based study was conducted in watermelon [*Citrullus lanatus *(Thunb.) Matsum. & Nakai var. lanatus] in order to elucidate the flow of events associated with fruit development and ripening in this species. RNA from three different maturation stages of watermelon fruits, as well as leaf, were collected from field grown plants during three consecutive years, and analyzed for gene expression using high-density photolithography microarrays and quantitative PCR.

**Results:**

High-density photolithography arrays, composed of probes of 832 EST-unigenes from a subtracted, fruit development, cDNA library of watermelon were utilized to examine gene expression at three distinct time-points in watermelon fruit development. Analysis was performed with field-grown fruits over three consecutive growing seasons. Microarray analysis identified three hundred and thirty-five unique ESTs that are differentially regulated by at least two-fold in watermelon fruits during the early, ripening, or mature stage when compared to leaf. Of the 335 ESTs identified, 211 share significant homology with known gene products and 96 had no significant matches with any database accession. Of the modulated watermelon ESTs related to annotated genes, a significant number were found to be associated with or involved in the vascular system, carotenoid biosynthesis, transcriptional regulation, pathogen and stress response, and ethylene biosynthesis. Ethylene bioassays, performed with a closely related watermelon genotype with a similar phenotype, i.e. seeded, bright red flesh, dark green rind, etc., determined that ethylene levels were highest during the green fruit stage followed by a decrease during the white and pink fruit stages. Additionally, quantitative Real-Time PCR was used to validate modulation of 127 ESTs that were differentially expressed in developing and ripening fruits based on array analysis.

**Conclusion:**

This study identified numerous ESTs with putative involvement in the watermelon fruit developmental and ripening process, in particular the involvement of the vascular system and ethylene. The production of ethylene during fruit development in watermelon gives further support to the role of ethylene in fruit development in non-climacteric fruits.

## Background

*Cucurbit *species, including watermelon [*Citrullus lanatus *(Thunb.) Matsum. & Nakai var. *lanatus*], produce large edible fruits that serve as an important component in the diets of people throughout the world [1]. In fact, watermelon accounts for 2% of the world's total area devoted to vegetable production [2]. In the United States, watermelon is considered an extremely important agricultural crop, with over 4.2 billion pounds being produced in 2006 and a fresh market value of $434 million [3].

Although there is narrow genetic diversity among watermelon cultivars [4], watermelon fruits are diverse in shape, size, rind thickness/color, in addition to flesh texture and color, sugar content, carotenoid and flavonoid composition (and associated aroma and flavor), and nutrient composition. There is considerable interest by seed companies and watermelon growers in enhancing watermelon fruit quality and nutritional values to address consumer desires. Like fruits of most plant species, the watermelon fruit undergoes sequential and rapid events during development and ripening [5,6]. Early fruit development involves rapid cell division, followed by a long phase of cell expansion to form large vacuolated cells that make up the flesh of watermelon fruits [7]. Cell expansion involves changes in cell wall structure and continuous accumulation (in the vacuoles) of carbohydrates, organic acids, and different compounds needed to retain the osmotic pressure and flow of water into the expanding cells [8]. During fruit ripening there are changes in pigments and aromatic volatiles, conversion of starch to sugars, and increased susceptibility to post-harvest pathogens [9]. The structural, biochemical, and physiological events occurring during the cell expansion and ripening phase make up the flavor, texture, and overall attractiveness of the ripe fruits.

Ripening is influenced by hormones, light, temperature, and developmental gene regulation [10]. Numerous studies have been conducted on a variety of plant species with respect to genes associated with cell wall metabolism, ethylene biosynthesis, and hormones affecting fruit set, growth, and ripening in both climacteric and non-climacteric fruits [11-16]. These studies report on coordinated expression of genes during growth and differentiation of the various tissues of the developing and ripening fruits. However, there is little information on genes controlling these processes in watermelon, a non-climacteric fruit [3]. Identifying, mapping, and characterizing these genes will be extremely useful to research and breeding efforts directed toward improvement of this crop.

In a recent study, we reported the construction of a subtracted and normalized cDNA library representing early fruit development (green flesh;12 days after pollination, (DAP), ripening stage (pink flesh; 24 DAP), and mature fruit (red flesh; 36 DAP) of watermelon [3]. In that study, we identified 832 EST-unigenes for watermelon fruit that can be classified, based on homology, into the following groups: metabolism, membrane transport, cytoskeleton synthesis and structure, cell wall formation and cell division, signal transduction, nucleic acid binding and transcription factors, defense and stress response, and secondary metabolism.

In this study, we utilize oligo-based microarrays and quantitative Real-Time PCR (Q-PCR) to identify genes modulated at differing stages of fruit development and ripening. Microarrays were composed of probes designed from 832 expressed sequence tags, derived from a fruit developmental cDNA library, which was normalized and subtracted against leaf [3]. These arrays were used to examine gene modulation during non-climacteric [17] fruit ripening at an early developmental stage (green flesh), early ripening (pink flesh), and in full ripe fruit (red flesh). A subset of these ESTs was further evaluated with quantitative-PCR. Biological replication for this work was performed over three consecutive years from field-grown watermelon fruits. The results of this study will serve as a basis for future investigations into genetic regulation of non-climacteric ripening in this important agricultural crop.

## Results/Discussion

### Microarray analysis

Microarray technology has proven to be an effective means for transcriptome analyses in developing and ripening fruits of different plant species, including strawberry [15,18] and tomato [19,20]. Microarray analysis of tomato facilitated the identification of new genes, such as transcription factors involved in fruit ripening, and genes controlling fruit quality including those involved in aroma, flavor, and pigmentation [19,20]. Cucurbits are distinct among dicotyledonous plants, primarily because of their ability to produce giant fruits with diverse shapes, sizes, aroma, flavors and colors. There is little information regarding expression of genes controlling fruit development and quality in cucurbit crops, including watermelon [3]. High-density photolithography-microarrays were used to profile 832 EST-unigenes that were previously identified from a subtractive watermelon fruit library [3]. The original subtractive library consisted of normalized and subtracted (against leaf) transcripts from all three time-points, thus differentiation of those transcripts from each individual time-point was not possible using the library alone. In addition, because of the nature of a subtractive library, transcript modulation, i.e. gene induction or repression, was not known. Of the 832 ESTs analyzed using microarrays, 335 (40%) were found to be modulated by at least two-fold (2x-ESTs) in at least one fruit tissue type, specifically early fruit development (green), fruit ripening (pink), and ripe (red) fruit collected in the first (Biorep1) of three growing seasons. At a 1.5-fold cut-off, 605 (73%) of the ESTs were differentially expressed in fruit development as compared to leaf tissue.

**Figure 1:**
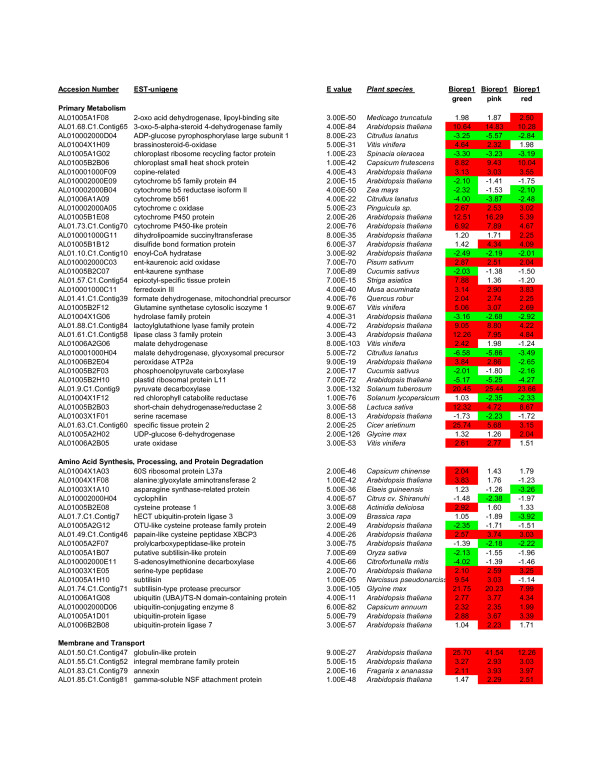
**List of 2x-ESTs that show at least two-fold differential expression by microarray analysis**. A total of 335 2x-ESTs were identified by microarray analysis that showed at least a two-fold difference in modulation as compared to leaf. These 2x-ESTs were compared to similar sequences in the Entrez database by blastx analysis and the strongest known homology was identified. This figure shows the upper quartile, for the full image please see Additional File 1. The 2x-ESTs induced at least two-fold were highlighted in red whereas 2x-ESTs repressed at least two-fold were highlighted in green. Microarray-determined fold-change is shown for each 2x-EST for green, pink, and red flesh as compared to leaf.

Of the 335 2x-ESTs identified by microarray analysis as modulated in developing fruits, 239 (71%) showed significant homology (e-values < 1 × 10^-5^) to genes discovered in other plant species or other organisms. Based on these homologies, 211 annotated 2x-ESTs can be classified into ten categories that include primary metabolism, amino acid synthesis, processing and protein degradation, membrane and transport, cell division, cytoskeleton, cell wall and metabolism, DNA and RNA related gene expression, signal transduction and defense and stress related genes (Figure 1; Additional File 1).

### Quantitative Real- Time PCR (Q-PCR)

**Figure 2:**
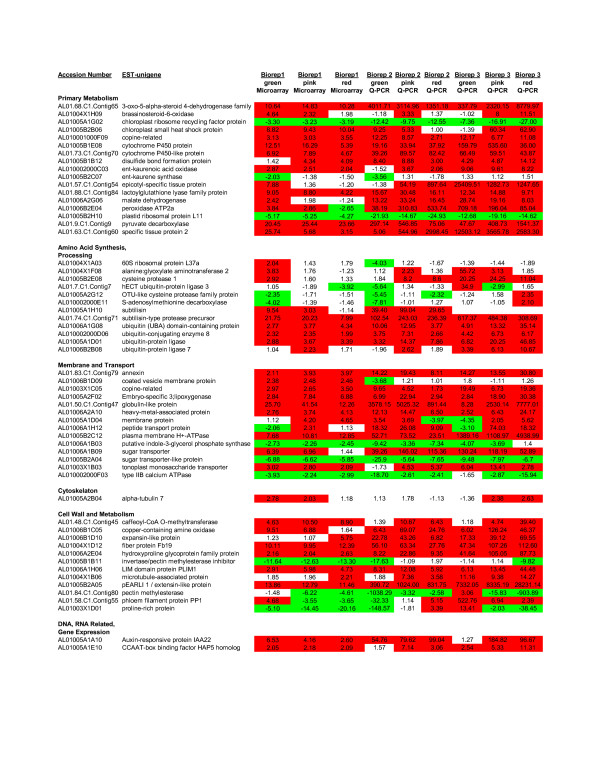
**Differential modulation of 127 2x-ESTs was confirmed by Q-PCR using two additional biological replicates**. One hundred and twenty seven 2x-ESTs that show differential modulation in the microarray (Biorep 1) were confirmed by Q-PCR (Biorep 2 & Biorep 3). This figure shows the first half of these 2x-ESTs, for the full image please see Additional File 2. The 2x-ESTs induced at least two-fold were highlighted in red whereas 2x-ESTs repressed at least two-fold were highlighted in green. Q-PCR fold inductions are shown for each 2x-EST for green, pink, and red flesh as compared to leaf.

To validate the microarray data, we performed Q-PCR on 127 2x-ESTs using all three fruit tissue types in comparison to leaf from the second and third growing seasons (Biorep 2 and Biorep 3, respectively) as shown in Figure 2 and Additional File 2. Ct values were compared between fruit tissues and leaf, and these data were compared to the microarray experiment using Biorep 1 and a 2-fold modulation cut-off. A high degree of correlation in terms of up- or down- regulation was found among all three Bioreps using the two detection methods. A total of 762 Q-PCR reactions of the three fruit tissue types and the two biological replicates (Biorep 2 & 3) were performed in duplicate. Twelve reactions failed in three trials leaving a total of 750 reactions. Seventy-two of the seven hundred and fifty (9.6%) tissue-type quantitative-reactions were in conflict with the microarray results, thus 90.4% were in agreement. When the individual fruit stages are considered separately, the Q-PCR results correlate well with the microarray results in terms of up or down regulation as follows: In early fruit (green fruit) 57.3% correlation between all Bioreps, 88% for ripening (pink fruit) across all Bioreps, and 72.7% for ripe (red fruit) across all Bioreps. When microarray results are compared against each individual Biorep, the results are as follows: in green fruit, correlation of microarray data to Q-PCR data of Biorep 2 and Biorep 3 is 81% and 71.8%, respectively; pink fruit is 88% and 91%, respectively; and red fruit is 75.8% and 85.9%, respectively.

### Overlapping and stage specific genes

**Figure 3:**
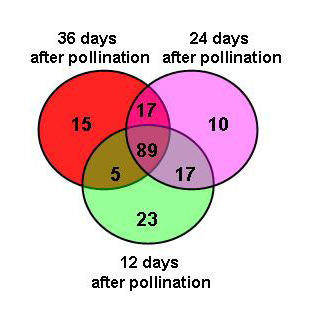
**Venn diagram of 176 induced 2x-ESTs**. Venn diagram showing the overlapping and stage-specific genes up-regulated in flesh tissue of watermelon fruit at three stages of development; green (12-days after pollination), pink (24-days after pollination) and red (36-days after pollination). Eighty-nine genes were expressed at all stages of development; some genes were stage specific (green 23; pink 10; and red 15) while others were overlapping in two stages (for gene identity see Figure 4 and Additional File 3).

**Figure 4:**
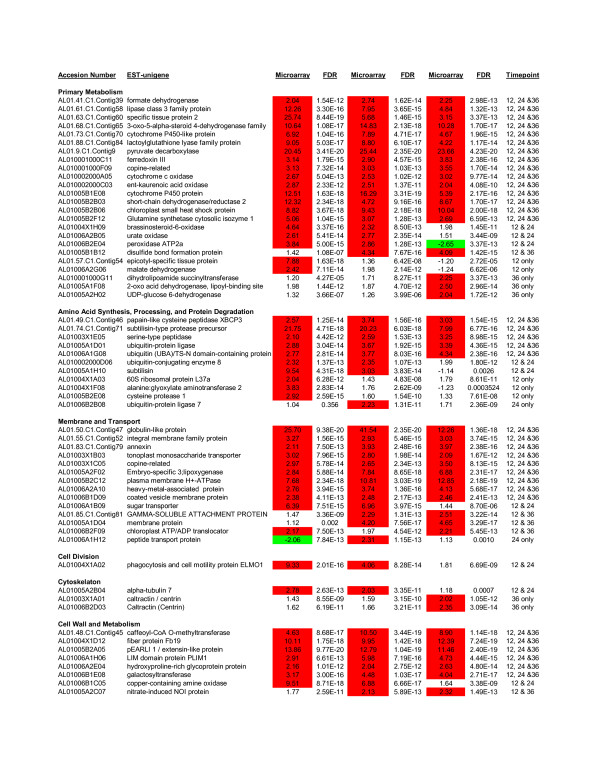
**The 2x-ESTs that are differentially modulated at a specific fruit stage compared to leaf as determined by microarray analysis**. One hundred and seventy-six 2x-ESTs that exhibit induction in one or more fruit types with a false discovery rate (FDR) of less than 0.05 were identified. This figure shows the first 60 of these 2x-ESTs, for the full image please see Additional File 3.

One hundred and seventy-six 2x-ESTs used in the microarray study were induced in one or more fruit stages compared to leaf (Figures 3 and 4; Additional File 3). Of these, the greatest number of shared 2x-ESTs between the fruit stages were those up-regulated in all three fruit stages (89 2x-ESTs). These up-regulated 2x-ESTs encode proteins with homology to four NAM transcription factors, six nodulin genes, two copine-related proteins, two proteins involved in production or response to ethylene (AL01006B1H12, AL01006B2H09), and four genes involved in programmed cell death (AL01006A1G08, AL010002000D06, AL01005A1D01, AL01005B2D01). Interestingly, 2x-ESTs that encode homologs for two caltractin-like proteins of which some members are involved in nucleotide excision repair or intercellular transport [21,22], are induced only at the ripe fruit stage.

One hundred and sixty-six 2x-ESTs were repressed in one or more fruit stages as compared to leaf (Figure 1; Additional File 1). Of these repressed 2x-ESTs, many encode homologs of proteins involved in the chloroplast, such as plastid Tic40, plastid transcriptionally active 17, chloroplast ribosome recycling factor, plastid ribosomal protein L11, and red chlorophyll catabolite reductase, as well as electron transfer such as cytochrome b5 family protein, cytochrome b5 reductase isoform II, and cytochrome b561.

### Ethylene production and watermelon fruit ripening

Many fruits classified as non-climacteric, such as citrus, grape, and strawberry, have been shown to produce ethylene during fruit ripening [23-25]. Once a climacteric fruit has reached full size, ripening begins through the production of internal ethylene, followed by softening of the cell walls, production of secondary compounds, as well as changes in sugar content, flavor, and aroma [3]. In contrast, non-climacteric fruits, by classical definition, do not exhibit the ripening -associated elevation in respiration typical of climacteric fruits. Most climacteric fruits also produce increased ethylene in concert with increased respiration. However, recent studies and microarray analysis of non-climacteric fruits indicate that ethylene and/or modulated sensitivity to ethylene might participate in physiological changes during non-climacteric fruit development [23-29]. Indeed, many non-climacteric fruits, including watermelon, are highly sensitive to exogenous ethylene [17].

Differential expression of homologs of genes involved in ethylene biosynthesis (ACC oxidase) and ethylene signal transduction [ethylene receptor Cm-ETR1, ethylene insensitive (EIN3/EIL)-like transcription factor, ethylene-responsive binding protein (EREBP), ethylene response factor (ERF)] was observed in our analysis. A homolog for ACC oxidase was induced in almost all fruit stages examined in watermelon. Also, a homolog for an ethylene receptor, Cm-ETR1 was induced in watermelon fruits. Strawberry exhibits an increase in ethylene receptor transcripts when the fruit matures from large green to white, concurrent with an increased synthesis of ethylene production [25]. This increase in ethylene production has also been seen in citrus, another non-climacteric fruit, where the young fruitlets produce relatively high ethylene levels that decrease at later developmental stages [24]. A homolog for an ethylene insensitive transcription factor (EIN3/EIL) was repressed in fruit stages examined in watermelon. This has also been shown in tomato where three orthologs, LeEIL1, LeEIL2, and LeEIL3 are all expressed at a higher level in leaf as compared to fruit [30]. A homolog for ERF was up-regulated in all three replicates for all watermelon fruit stages. A homolog for S-adenosylmethionine decarboxylase, an enzyme involved in polyamine biosynthesis, is highly down-regulated in green fruit in two replicates in watermelon. Ethylene and polyamine biosynthesis compete for S-adenosylmethionine (SAM) when its supply is low [31], thus it appears that competition for SAM may occur at the green stage of watermelon fruit development.

**Figure 5:**
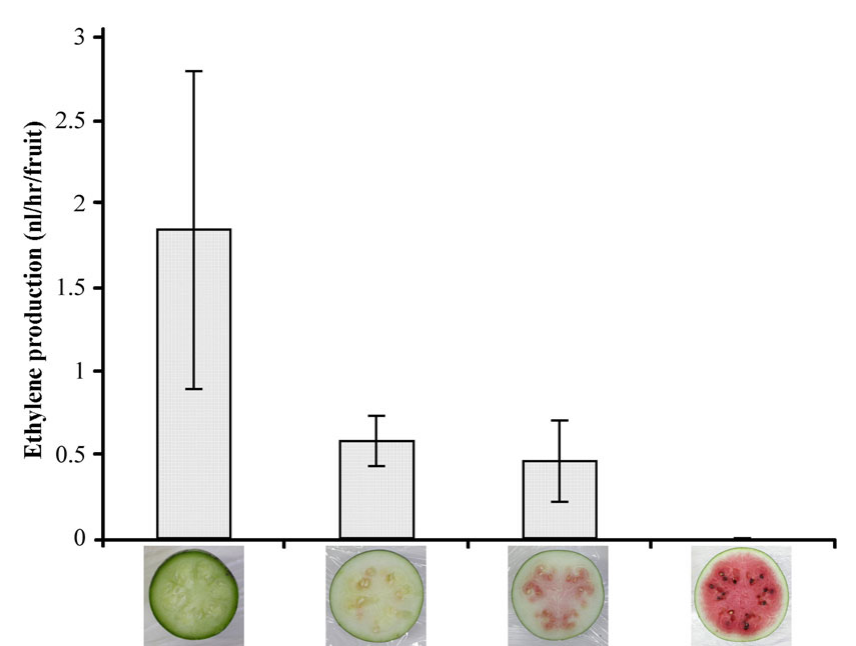
**Ethylene production during fruit development**. The watermelon cultivar 'Sugar Baby' was grown and ethylene production was measured during the green, white, pink and red fruit developmental stages.

To determine if ethylene is involved in fruit ripening in non-climacteric watermelon, we measured the amount of ethylene being produced in the watermelon cultivar 'Sugar Baby', one of the most popular red flesh watermelon cultivars [32]. At the green fruit stage, 'Sugar Baby' produced about 1.8 nl of ethylene/hr/fruit (Figure 5). As the fruit continued to develop, there was a marked decrease in ethylene production until, in the red fruit, ethylene synthesis is below detection level (Figure 5). Thus, from these data we show that ethylene production is highest in the green fruit stage of watermelon and decreases in later developmental stages, similar to citrus [24,25]. To our knowledge, this is the first time ethylene production has been measured in watermelon during fruit ripening. Our data, as well as EST-derived expression analyses of other non-climacteric fruits, highlight the need for re-examination of the role that ethylene may play in non-climacteric fruits [33].

### Vascular Development-Xylem

Tracheary elements (TE) are specialized cells in the xylem that transport water and solutes [34]. Normally derived from the procambium and cambium, TE differentiation can be divided into five stages of development: (1) early xylem differentiation, (2) cell expansion, (3) secondary cell wall deposition, followed by (4) programmed cell death (PCD), which leads to the formation of a (5) mature TE [34]. Of the watermelon 2x-ESTs identified in this study, 29 2x-ESTs have homologs that are associated with TE differentiation (Figure 1; Additional File 1).

Tracheary elements have been extensively studied using the *Zinnia elegans *mesophyll cell system. These studies have shown that genes expressed during the initial phases of TE differentiation (stage 1) include those involved in wound response, such as protease inhibitors, and those involved in protein synthesis, such as ribosomal proteins and elongation factor genes [29,35]. In addition, calcium and calmodulin are required during the early stages of TE formation [31]. Seven calcium or calmodulin binding EST homologs, including a homolog for calmodulin, are differentially regulated in watermelon during one or more of the developmental stages.

During the next phase of TE development (stage 2–3) in the *Zinnia *system, the cytoskeleton undergoes changes as tubulin transcripts increase, causing a corresponding increased in the number of microtubules [29]. These microtubules form bands that mark the sites where cellulose, lignin, hemicellulose, and proteins will be deposited to form the secondary cell wall [34]. The cellulose synthase complex and hemicelluloses are transported to these microtubule sites by vesicles [34]. The hormone gibberellin plays an important role in TE differentiation and lignification, as suppression of gibberellin biosynthesis in *Zinnia *results in suppression of lignification in TE cells [36]. A microtubule-associated protein homolog and a tubulin homolog were up-regulated during watermelon fruit development. Also, a homolog for Caffeoyl-CoA O-methyltransferase, an enzyme involved in lignin deposition in differentiating TEs in Zinnia [37], was highly up-regulated in the pink and red fruit stage of watermelon. Two genes homologous to those involved in the biosynthesis of gibberellins, ent-kaurene synthase and ent-kaurenoic acid oxidase, were differentially regulated in developing and ripening watermelon fruits.

Brassinolides have been shown to be required before secondary cell wall formation and autolysis occur in tracheary cells [35]. *Arabidopsis *brassinolide biosynthesis mutants such as cpd, dwarf7, and det2 have fewer xylem cells than wild-type [34]. In watermelon, 3-oxo-5-alpha-steroid 4-dehydrogenase and brassinosteroid-6-oxidase, both enzymes involved in brassinolide biosynthesis, are highly up-regulated in most watermelon stages examined.

In the maturing TE, after the secondary cell wall is formed, the vacuole collapses and releases proteases, DNAses, and RNAses, at which point PCD occurs (stage 4) [34]. Genes involved with PCD were highly up-regulated during all stages of development and fruit ripening in watermelon. This can be an indication that continuous TE formation is an integral part of watermelon fruit development. Two putative ubiquitin ligases (AL01005A1D01, AL01006B2B08), one ubiquitin-conjugating enzyme homolog, as well as an ubiquitin were induced in nearly all watermelon fruit development stages. Two cysteine protease/peptidase homologs (AL01005B2E08, AL01005A2G12) and two subtilisins were induced in at least one fruit developmental stage in our study. The increased activity of protein degradation machinery is characteristic of PCD and supports our hypothesis that TE formation is an inherent trait of watermelon fruit development.

Transcription factors that regulate xylogenesis have been identified in previous studies, among these are the NAM, ATAF, and CUC (NAC) family of transcription factors, of which several have been found to be preferentially expressed in developing wood, differentiating TEs, and during secondary cell wall thickening [38]. In *Arabidopsis*, seven vascular-related NAC-domain (VND) genes were preferentially expressed during xylogenesis of which two, VND6 and VND7, have been further characterized to show that ectopic expression of these transcription factors can cause trans-differentiation of differentiated cells into metaxylem- and protoxylem- like vessel cells [38]. Furthermore, ectopic expression of the NAC secondary wall thickening promoting factor (NST) 1 and 2 genes in *Arabidopsis *resulted in ectopic secondary cell wall thickening in various tissues and epidermal cells with ectopic thickening had structural features similar to TEs [39]. In our study, three NAC protein homologs were found to be consistently up-regulated in developing and ripening watermelon fruits (AL01.82.C1.Contig78, AL01.70.C1.Contig67, AL01004X1E11). We conjecture that these transcription factors may play a similar role in vascular differentiation as has been shown in other species [38].

### Vascular Development-Phloem

Six nodulin protein homologs were up-regulated during fruit development and ripening in watermelon (AL01.0.C1.Contig1, AL01.69.C1.Contig66, AL01005A2B11, AL01003X1E03, AL01006A1C09, AL01006A2D05). Other fruit ripening studies have reported similar differential expression of nodulin-related genes despite these plants being non-symbiotic [40-42]. A recent study in *Arabidopsis *identified an early nodulin-like protein that localizes to the plasma membrane of differentiating and mature sieve elements [43]. Sieve elements are the conductive cells of the phloem. It is possible that these up-regulated nodulin genes may play a role in phloem function in watermelon.

The phloem sieve elements contain proteinacious filaments and aggregates called P-proteins [44]. In Cucurbits, the phloem filaments are composed of phloem filament protein PP1 and the phloem lectin PP2. In our study, a homolog for the phloem filament protein PP1 (AL01.58.C1.Contig55) was differentially regulated in developing and ripening watermelon fruits in all three replicates. Besides the structural proteins, the phloem translocation stream has an antioxidant defense system, thought to provide defense against reactive oxygen species [45]. In accordance, the antioxidant protein peroxidase (AL01006B2E04) is up-regulated in all three bioreps in the green and pink fruit stages.

The process called phloem unloading allows sugars to be moved from sieve elements to recipient sink cells in the developing fruit [46]. These sink cells enlarge as H+ ATPase and H+ pyrophosphatase drives transport of sugars, minerals, and organic acids into the vacuole [47]. Accordingly, increased expression was seen in homologs of genes involved in sugar transport such as plasma membrane H+ATPase (AL01005B2C12), a sugar transporter (AL01006A1B09), and a tonoplast monosaccharide transporter (AL01003X1B03) in developing and ripening watermelon fruits.

The connection between fruit development and ripening and the development of the vascular system was seen in a similar microarray study of fruit-ripening in strawberry [29]. The development of a vascular system in fleshy fruits is important, as the fruit serves as a nutrient sink and the vascular system provides the framework for water (which constitutes 92% of the watermelon fruit), nutrients, and sugars to flow from the vegetative parts to the developing fruit of the plant. Thus, the rapid fruit expansion and growth in watermelon is likely due to the development of the vascular system.

### Fruit expansion

Watermelon fruits develop and mature rapidly [3]. Thus, it is not surprising that many of the 2x-ESTs identified in this study are involved in or associated with the expanding cell wall. Expansins, which are cell wall loosening factors, have homologs in watermelon that are highly up-regulated. The cell wall proteins which include homologs of proline-rich proteins, hydroxyproline-rich glycoproteins, and extensins show differential regulation in developing and ripening watermelon fruits. Three homologs of auxin-related genes, indole-3-glycerol phosphate synthase, auxin-repressed protein ARP1, and auxin-responsive protein IAA22 were differentially expressed in watermelon fruits. The hormone auxin is well known to regulate cell growth and to induce tracheary element differentiation [31,35].

### Secondary metabolism

Carotenoids, including lycopene which imparts the red color to watermelon, and flavonoids are secondary metabolites in watermelon fruits as well as in other fruits such as tomato [3,48]. Homologs for phytoene synthase and phytoene desaturase, which are enzymes in the carotenoid biosynthetic pathway, are repressed in almost all developmental stages of watermelon. Two phytoene synthase genes, PSY-1 and PSY-2, have been identified in tomato. PSY-1 is responsible for carotenoid biosynthesis during fruit ripening and PSY-2 is responsible for carotenoid formation in chloroplast containing tissues. Of these two genes, only PSY-2 is repressed in ripening fruits when compared to leaf [49,50]. It is possible that the homolog for phytoene synthase in watermelon has a function similar to PSY-2 in tomato.

### Signal transduction/transcription

Ten homologs for transcription factors were found to be modulated in watermelon fruits at different developmental stages. These regulatory genes include basic region/leucine zipper (bZIP) proteins, zinc finger proteins, a CCAAT box binding factor protein, a MYB transcription factor, and a MADS box transcription factor. Other transcription factors, such as those involved in MADS box regulation of fruit ripening, have been implicated in both climacteric (tomato) and non-climacteric (strawberry) fruit ripening [51].

### Defense and stress related

The ability of fruit to resist pathogen attack and environmental stress decreases with fruit ripening [3,29]. Quiescent pathogens such as *Botrytis *and *Alternaria*, located on or in the fruit, initiate necrotrophic development in ripening fruits [52]. Many gene homologs involved in resistance, pathogenesis, and stress, such as *Citrus tristeza virus *resistance gene, powdery mildew resistance, pathogenesis protein, silverleaf whitefly-induced proteins, DNAJ heat shock proteins, and harpin-induced proteins, show differential expression in developing and ripening watermelon fruits. Because the Bioreps were taken from field conditions over three different years, the possibility exists that the fruits were responding to some form of pathogen attack. A 2x-EST with homology to the PMR5 powdery mildew resistance gene was found to be highly down regulated in comparison to leaf tissue in all Bioreps and all developmental stages. The powdery mildew pathogens, *Sphaerotheca fuliginea *(Schlechtend.:Fr.) Pollacci and *Erysiphe cichoracearum *DC., are ubiquitous throughout the Unites States, and can easily be found in most watermelon fields during most times of the growing season. This is a foliar pathogen and thus it would make sense that the leaves would be expressing this gene more than the fruit. In contrast, the 2x-EST homolog of the silverleaf whitefly-induced protein is highly induced in all fruit stages during each sampling year. It would be unlikely that this increase in expression level to be in response to a whitefly attack due to this pest's preference to the foliar portions and stems of the plant as opposed to the fruit. This increase is more likely in response to environmental stresses that are common in field grown fruit crops. Many pathogenesis related genes and resistance pathways are closely associated with the stress pathways in a plant, thus increased expression patterns in a resistance gene may not be directly related to a specific pathogen.

## Conclusion

In this study we identified 335 2x-ESTs with differential expression in developing and ripening watermelon fruits when compared to leaf. Validation of microarray results with Q- PCR using 127 2x-ESTs showed a high similarity of modulation between bioreps. These 2x-ESTs comprise a range of putative functions including metabolism related to fruit ripening, aspects of hormone synthesis and signalling, pathogen response, cell wall modification and general transcriptional control. A considerable number of genes associated with the vascular system are modulated in the developing and ripening watermelon fruit. Additionally, this is the first report that provides data that ethylene may be involved in developing and ripening of these "non-climacteric" fruits. Along these lines we show that watermelon produces the most ethylene during the green fruit stage with decreased production of ethylene during the white and pink fruit stages. The ripening-associated watermelon ESTs described here represents a foundation for further characterization of fruit development and ripening in this important cucurbit species.

## Methods

### Plant Material

Watermelon fruits at green flesh stage (12 DAP), pink flesh stage (24 DAP), and red flesh stage (36 DAP) from the cultivar 'Illini Red' were used for RNA isolation. In addition, several leaves in different stages of development, immature (not fully expanded), young (fully expanded), and mature, were collected from the experimental plants, combined, and processed for RNA isolation as described below. The watermelon plants were grown in a field plot located at the South Central Agricultural Research Laboratory at Lane, OK. Upon collection, fruits were rinsed with sterile de-ionized water in the field, followed by flesh-tissue excision and processing as previously described [3,53]. Three separate, biological replications (Biorep 1–3) of fruit and leaf were performed over a three year period and used in the described microarray and Q-PCR studies.

### RNA Isolation

Total RNA was isolated from watermelon fruits and leaves from all bioreps as previously described [3,53]. RNA quality and quantity were determined using a spectrophotometer, denaturing agarose gel electrophoresis [54] and an Agilent 2100 Bioanalyzer (Agilent Technologies, Santa Clara, CA). Only RNAs with an OD_260_:OD_280 _ratio of >1.80 and no discernable degradation were used in microarray or PCR-based experiments.

### RNA Clean-up

RNAs from all bioreps of the three stages of developing fruit and leaf, as described above, were processed to remove any contaminating genomic DNA as follows: 100 μg of each RNA was treated in a 100 μl DNaseI cocktail consisting of 10 μl 10× RQ1 buffer (Promega, Madison, WI), 5 μl RQ1 RNase-free DNAseI (Promega) and RNAse-free H_2_O (Gibco/Invitrogen, Carlsbad, CA). This reaction was performed at 37°C for 30 min, followed by the addition of 5 μl Stop Solution [20 mM EGTA, pH8.0 (Promega)], then placed at 65°C for 10 min. The entire reaction was then run through the RNA clean-up protocol using the Qiagen RNeasy kit (Qiagen, Inc., Valencia, CA).

### Microarray design and production

Eight-hundred and thirty two EST-unigenes associated with watermelon fruit development were previously identified and described [3]. These EST-unigenes, from a normalized and subtracted cDNA library representing three distinct time points in fruit development; green stage, pink stage, and red stage were used for the basis of the gene expression analysis in this study. Each of the 832 EST-unigenes was used by NimbleGen Systems (Madison, WI) to design and manufacture high-density photolithography-microarrays. A single microarray chip containing twelve, independently-hybridisable, identical mini-arrays [3 technical replicates × 4 tissue type/developmental stages (green, pink, and red, and leaf)] was utilized for this study. Each of the 12 mini-arrays contained ~12,500 probes representing 832 EST-unigenes. Each EST-unigene was represented by a minimum of fifteen 24-mer probes. Probes were synthesized *in situ *by photolithography on glass slides using a computer-generated randomized pattern on the array. RNA from a single biological replicate, Biorep 1, was used for the microarray experiment. cDNA from each tissue type was labelled and hybridized to three individual mini-arrays, thus three technical replications were performed for each RNA sample. A subset of identified, modulated genes from this experiment was used in quantitative-PCR for Biorep 2 and 3 RNAs, which are described below.

### Hybridization, Cy-2 conjugation and antibody amplification

RNA samples from flesh tissue of watermelon fruits at the green, pink and red stage, as well as from leaf were provided to NimbleGen Systems, Inc. All hybridizations, staining, and processing of arrays were performed by NimbleGen Systems.

### Array scanning, data extraction, normalization, analysis, and deposition of data

Scanning, data extraction, and array calibrations were performed by NimbleGen Systems, Inc. Each array was scanned using an Axon 4000 scanner at a PMT setting that allows all features to be slightly below saturation. These data were extracted using the NimbleScan software. This extraction included an initial pass to identify possible outliers in the probe sets. Outlier probes were flagged and removed from further calculations. In addition, probe that were more than three standard deviations from the mean of the probe set were also flagged. Any probe flagged and omitted from one data set, was also eliminated from the other technical replicate sets as well so that all calculations for each gene were performed using the same set of data points. Normalization of the data, which attempts to remove variation within and across arrays, was performed using the Robust Multichip Average (RMA) method at the probe level [55,56]. Differentially expressed genes were identified using LIMMA [57], and multiple test correction of raw p-values was performed using the False Discovery Rate (FDR) [58]. 2x-ESTs with a fold change of at least two, when gene expression in fruit was compared to that in leaf, and FDR less than 0.05, were identified as differentially expressed. All identified differentially expressed genes were clustered using the *k*-means algorithm implemented in Cluster 3.0 software package [59]. All microarray data from these experiments have been deposited into the National Center for Biotechnology Information (NCBI) Gene Expression Omnibus (GEO) [60] and are accessible through GEO series accession number GSE11246.

### Oligonucleotide design for Q-PCR

Primer sets for a total of 127 different ESTs (Additional File 4) were designed with PrimerQuest^© ^software (International DNA Technologies (IDT), Inc., Coralville, IA). The oligonucleotides were synthesized by IDT. Primer sets were used in quantitative-PCR (Q-PCR) studies described below.

### Quantitative Real-Time PCR

cDNA was generated using 3 μg of the purified RNA described above. The Invitrogen SuperScript™ III First-strand synthesis system for RT-PCR was used according to the manufacturer protocol and the Oligo(dT)_20 _(50 mM) primer supplied with the kit was used for reverse transcription (Invitrogen, La Jolla, CA). cDNA concentrations were quantified using a Biophotometer (Eppendorf, Westbury, NY) or an Agilent 2100 Bioanalyzer (Agilent Technologies, Santa Clara, CA). Q-PCR reactions were run in a Stratagene Mx3000P Real-Time PCR system (Stratagene, La Jolla, CA). Each 25 μL reaction consisted of 0.5 ng cDNA, 5 μL of primer mix (1.5 μM of each forward and reverse primer) (additional file 1), 12.5 μL of Brilliant^® ^Sybr^® ^Green Master Mix (Stratagene, Cedar Creek, TX), 0.375 μL ROX reference dye and H_2_O. Template DNA from the watermelon "Illini Red" was used to determine optimal Q-PCR reaction conditions for each primer pair. Individual reactions were run with each primer pair with annealing temperatures ranging from 57°C to 63°C. Cycling conditions were 95°C for 10 min, followed by 40 cycles of: 95°C for 30 sec, gradient from 57°C to 63°C, and 72°C for 30 sec. Dissociation curves were performed at the end of each reaction run to detect primer-dimer and secondary products. All Q-PCR runs were performed using the above cycling conditions and times with the exception of using a single annealing temperature of 60°C. cDNA from each tissue type: early development stage fruits, ripening stage fruits, ripe fruits, and from leaf, were run in duplicate on the Real-Time system. Quantification was achieved by normalizing the number of target gene copies to an endogenous reference gene by using the comparative Ct method [61]. The ΔCt was calculated by subtracting the average Ct value of each tissue type from the average Ct values of 18S rRNA (ribosomal primers obtained from Applied Biosystems, Foster City, CA). The ΔΔCt was calculated by subtracting the ΔCt of each of the three fruit stages from the ΔCt of the leaf tissue. The formula 2 ^-(ΔΔCt) was used to calculate a relative fold change between the leaf and the fruit. This relative fold change was determined by assuming a near perfect amplification resulting in a doubling of amplification product per cycle.

### Measurement of ethylene production

Ethylene production was determined in watermelon fruits of the 'Sugar Baby' cultivar at four developmental stages: fruitlets (7 grams), young fruit (483 grams), ripening fruits (1,660 grams) and ripe fruits (3,266 grams). Fruits were sealed for 4 h in a gas-tight nylon bag equipped with a closed syringe fitted with a serum cap. The air was emptied from the fruit-containing bag with a vacuum pump and 50 ml of fresh air was injected into the bag through the serum cap [24]. Two-ml samples were withdrawn from the bag with a hypodermic syringe and injected into a Varian 3300 gas chromatograph equipped with an alumina column at 100°C and detected using a flame ionization detector (FID) at 120°C.

## Authors' contributions

WPW and AL conceived the experimental design and objectives of all the microarray and Q-PCR experiments, conducted the microarray and Q-PCR analyses, and wrote the manuscript. KRH conducted Q-PCR and microarray data analyses and took an active part in writing the manuscript. ARD planted and grew the watermelons and isolated RNA from watermelon fruits in sequential developmental stages for microarray and Q-PCR analyses, and took part in reviewing and writing the manuscript. AH and JT were responsible for the construction of the cDNA library for watermelon, sequencing and contig generation of the ESTs, bioinformatics of the ESTs, and critical reviews of the manuscript. ZF performed the bioinformatics and analysis of all microarray data. TT and AS-M designed and performed experiments with ethylene related genes and preparation of the manuscript. YT, NK, VP, and JJG participated in design and implementation of the microarray and Q-PCR experiments related to this study and took part in reviewing and writing this manuscript. All authors have read and approved the manuscript.

## Supplementary Material

Additional file 1 **List of 335 2x-ESTs that show at least two-fold differential expression by microarray analysis**. Three hundred thirty-five 2x-ESTs were identified by microarray analysis that showed at least a two-fold difference in modulation when compared to leaf. 2x-ESTs induced at least two-fold were highlighted in red whereas 2x-ESTs repressed at least two-fold were highlighted in green. Microarray fold inductions are shown for each 2x-EST for green, pink, and red flesh as compared to leaf. NA = Not applicable. Click here for file

Additional file 2 **Differential modulation of 127 2x-ESTs was confirmed by Q-PCR using two additional biological replicates**. One hundred twenty-seven 2x-ESTs that show differential modulation in the microarray (Biorep 1) were confirmed by Q-PCR (Biorep 2 & Biorep 3). 2x-ESTs induced at least two-fold were highlighted in red whereas 2x-ESTs repressed at least two-fold were highlighted in green. Q-PCR fold inductions are shown for each 2x-EST for green, pink, and red flesh as compared to leaf. Click here for file

Additional file 3 **2x-ESTs that are differentially modulated at a specific fruit stage compared to leaf as determined by microarray analysis**. One hundred and seventy-six 2x-ESTs that exhibit induction in one or more fruit types with a false discovery rate (FDR) of less than 0.05 were identified. Click here for file

Additional file 4 Primer sets and amplicon size for 2x-ESTs used in Q-PCR. Click here for file
