# Supplementary material for: Gene expression in developing watermelon fruit
Source: BMC Genomics. 2008 Jun 5;9:275. doi: 10.1186/1471-2164-9-275 (PMC2440768; doi:10.1186/1471-2164-9-275)
Supplement: Additional file 1 — List of 335 2x-ESTs that show at least two-fold differential expression by microarray analysis. Three hundred thirty-five 2x-ESTs were identified by microarray analysis that showed at least a two-fold difference in modulation when compared to leaf. 2x-ESTs induced at least two-fold were highlighted in red whereas 2x-ESTs repressed at least two-fold were highlighted in green. Microarray fold inductions are shown for each 2x-EST for green, pink, and red flesh as compared to leaf. NA = Not applicable. [file 1471-2164-9-275-S1.pdf]

Accesion Number	EST-unigene	E value	Plant species	Biorep1 green	Biorep1 pink	Biorep1 red
**Primary Metabolism**						
AL01005A1F08	2-oxo acid dehydrogenase, lipoyl-binding site	3.00E-50	*Medicago truncatula*	1.98	1.87	2.50
AL01.68.C1.Contig65	3-oxo-5-alpha-steroid 4-dehydrogenase family	4.00E-84	*Arabidopsis thaliana*	10.64	14.83	10.28
AL010002000D04	ADP-glucose pyrophosphorylase large subunit 1	8.00E-23	*Citrullus lanatus*	-3.25	-5.57	-2.84
AL01004X1H09	brassinosteroid-6-oxidase	5.00E-31	*Vitis vinifera*	4.64	2.32	1.98
AL01005A1G02	chloroplast ribosome recycling factor protein	1.00E-23	*Spinacia oleracea*	-3.30	-3.23	-3.19
AL01005B2B06	chloroplast small heat shock protein	1.00E-42	*Capsicum frutescens*	8.82	9.43	10.04
AL010001000F09	copine-related	4.00E-43	*Arabidopsis thaliana*	3.13	3.03	3.55
AL010002000E09	cytochrome b5 family protein #4	2.00E-15	*Arabidopsis thaliana*	-2.10	-1.41	-1.75
AL010002000B04	cytochrome b5 reductase isoform II	4.00E-50	*Zea mays*	-2.32	-1.53	-2.10
AL01006A1A09	cytochrome b561	4.00E-22	*Citrullus lanatus*	-4.00	-3.87	-2.48
AL010002000A05	cytochrome c oxidase	5.00E-23	*Pinguicula sp.*	2.67	2.53	3.02
AL01005B1E08	cytochrome P450 protein	2.00E-26	*Arabidopsis thaliana*	12.51	16.29	5.39
AL01.73.C1.Contig70	cytochrome P450-like protein	2.00E-76	*Arabidopsis thaliana*	6.92	7.89	4.67
AL010001000G11	dihydrolipoamide succinyltransferase	8.00E-35	*Arabidopsis thaliana*	1.20	1.71	2.25
AL01005B1B12	disulfide bond formation protein	6.00E-37	*Arabidopsis thaliana*	1.42	4.34	4.09
AL01.10.C1.Contig10	enoyl-CoA hydratase	3.00E-92	*Arabidopsis thaliana*	-2.49	-2.19	-2.01
AL010002000C03	ent-kaurenoic acid oxidase	7.00E-70	*Pisum sativum*	2.87	2.51	2.04
AL01005B2C07	ent-kaurene synthase	7.00E-89	*Cucumis sativus*	-2.03	-1.38	-1.50
AL01.57.C1.Contig54	epicotyl-specific tissue protein	7.00E-15	*Striga asiatica*	7.88	1.36	-1.20
AL010001000C11	ferredoxin III	4.00E-40	*Musa acuminata*	3.14	2.90	3.83
AL01.41.C1.Contig39	formate dehydrogenase, mitochondrial precursor	4.00E-76	*Quercus robur*	2.04	2.74	2.25
AL01005B2F12	Glutamine synthetase cytosolic isozyme 1	9.00E-67	*Vitis vinifera*	5.06	3.07	2.69
AL01004X1G06	hydrolase family protein	4.00E-31	*Arabidopsis thaliana*	-3.16	-2.68	-2.92
AL01.88.C1.Contig84	lactoylglutathione lyase family protein	4.00E-72	*Arabidopsis thaliana*	9.05	8.80	4.22
AL01.61.C1.Contig58	lipase class 3 family protein	3.00E-43	*Arabidopsis thaliana*	12.26	7.95	4.84
AL01006A2G06	malate dehydrogenase	8.00E-103	*Vitis vinifera*	2.42	1.98	-1.24
AL010001000H04	malate dehydrogenase, glyoxysomal precursor	5.00E-72	*Citrullus lanatus*	-6.58	-5.86	-3.49
AL01006B2E04	peroxidase ATP2a	9.00E-19	*Arabidopsis thaliana*	3.84	2.86	-2.65
AL01005B2F03	phosphoenolpyruvate carboxylase	2.00E-17	*Cucumis sativus*	-2.01	-1.80	-2.16
AL01005B2H10	plastid ribosomal protein L11	7.00E-72	*Arabidopsis thaliana*	-5.17	-5.25	-4.27
AL01.9.C1.Contig9	pyruvate decarboxylase	3.00E-132	*Solanum tuberosum*	20.45	25.44	23.66
AL01004X1F12	red chlorophyll catabolite reductase	1.00E-76	*Solanum lycopersicum*	1.03	-2.35	-2.33
AL01005B2B03	short-chain dehydrogenase/reductase 2	3.00E-58	*Lactuca sativa*	12.32	4.72	8.67
AL01003X1F01	serine racemase	8.00E-13	*Arabidopsis thaliana*	-1.73	-2.23	-1.72
AL01.63.C1.Contig60	specific tissue protein 2	2.00E-25	*Cicer arietinum*	25.74	5.68	3.15
AL01005A2H02	UDP-glucose 6-dehydrogenase	2.00E-126	*Glycine max*	1.32	1.26	2.04
AL01006A2B05	urate oxidase	3.00E-53	*Vitis vinifera*	2.61	2.77	1.51
**Amino Acid Synthesis, Processing, and Protein Degradation**						
AL01004X1A03	60S ribosomal protein L37a	2.00E-46	*Capsicum chinense*	2.04	1.43	1.79
AL01004X1F08	alanine:glyoxylate aminotransferase 2	1.00E-42	*Arabidopsis thaliana*	3.83	1.76	-1.23
AL01003X1A10	asparagine synthase-related protein	5.00E-36	*Elaeis guineensis*	1.23	-1.26	-3.26
AL010002000H04	cyclophilin	4.00E-57	*Citrus cv. Shiranuhi*	-1.48	-2.38	-1.97
AL01005B2E08	cysteine protease 1	3.00E-68	*Actinidia deliciosa*	2.92	1.60	1.33
AL01.7.C1.Contig7	hECT ubiquitin-protein ligase 3	3.00E-09	*Brassica rapa*	1.05	-1.89	-3.92
AL01005A2G12	OTU-like cysteine protease family protein	2.00E-49	*Arabidopsis thaliana*	-2.35	-1.71	-1.51
AL01.49.C1.Contig46	papain-like cysteine peptidase XBCP3	4.00E-26	*Arabidopsis thaliana*	2.57	3.74	3.03
AL01005A2F07	prolylcarboxypeptidase-like protein	3.00E-75	*Arabidopsis thaliana*	-1.39	-2.18	-2.22
AL01005A1B07	putative subtilisin-like protein	7.00E-69	*Oryza sativa*	-2.13	-1.55	-1.96
AL010002000E11	S-adenosylmethionine decarboxylase	4.00E-66	*Citrofortunella mitis*	-4.02	-1.39	-1.46
AL01003X1E05	serine-type peptidase	2.00E-70	*Arabidopsis thaliana*	2.10	2.59	3.25
AL01005A1H10	subtilisin	1.00E-05	*Narcissus pseudonarciss*	9.54	3.03	-1.14
AL01.74.C1.Contig71	subtilisin-type protease precursor	3.00E-105	*Glycine max*	21.75	20.23	7.99
AL01006A1G08	ubiquitin (UBA)/TS-N domain-containing protein	4.00E-11	*Arabidopsis thaliana*	2.77	3.77	4.34
AL010002000D06	ubiquitin-conjugating enzyme 8	6.00E-82	*Capsicum annuum*	2.32	2.35	1.99
AL01005A1D01	ubiquitin-protein ligase	5.00E-79	*Arabidopsis thaliana*	2.88	3.67	3.39
AL01006B2B08	ubiquitin-protein ligase 7	3.00E-57	*Arabidopsis thaliana*	1.04	2.23	1.71
**Membrane and Transport**						
AL01.50.C1.Contig47	globulin-like protein	9.00E-27	*Arabidopsis thaliana*	25.70	41.54	12.26
AL01.55.C1.Contig52	integral membrane family protein	5.00E-15	*Arabidopsis thaliana*	3.27	2.93	3.03
AL01.83.C1.Contig79	annexin	2.00E-16	*Fragaria x ananassa*	2.11	3.93	3.97
AL01.85.C1.Contig81	gamma-soluble NSF attachment protein	1.00E-48	*Arabidopsis thaliana*	1.47	2.29	2.51

AL010001000G01	DUF895 domain protein	1.00E-16	*Aspergillus clavatus*	1.28	-2.43	-2.14
AL010002000A08	aquaporin	2.00E-82	*Cucurbita ficifolia*	-3.41	-2.63	-3.88
AL010002000F03	type IIB calcium ATPase	4.00E-30	*Medicago truncatula*	-3.93	-2.24	-2.99
AL01003X1B03	tonoplast monosaccharide transporter	5.00E-65	*Arabidopsis thaliana*	3.02	2.80	2.09
AL01003X1C05	copine-related	2.00E-16	*Arabidopsis thaliana*	2.97	2.65	3.50
AL01005A1D04	membrane protein	7.00E-58	*Oryza sativa*	1.12	4.20	4.65
AL01005A2F02	Embryo-specific 3;lipoxygenase	4.00E-48	*Medicago truncatula*	2.84	7.84	6.88
AL01005B1B05	Pm52	3.00E-14	*Prunus mume*	-1.13	-1.73	-2.17
AL01005B1D10	sulphate transporter	1.00E-13	*Brassica napus*	-4.40	-3.37	-5.01
AL01005B1G06	endomembrane protein 70	1.00E-12	*Arabidopsis thaliana*	-1.64	-2.53	-2.55
AL01005B2A04	sugar transporter-like protein	5.00E-26	*Nicotiana langsdorffii*	-6.88	-6.62	-5.85
AL01005B2C12	plasma membrane H+-ATPase	5.00E-09	*Daucus carota*	7.68	10.81	12.85
AL01006A1B03	putative indole-3-glycerol phosphate synthase	4.00E-22	*Arabidopsis thaliana*	-2.73	-2.26	-2.45
AL01006A1B09	sugar transporter	4.00E-37	*Oryza sativa*	6.39	6.96	1.44
AL01006A1C01	MRP-like ABC transporter	1.00E-46	*Oryza sativa*	-6.80	-2.79	-4.07
AL01006A1C11	sulphate transporter	6.00E-52	*Populus alba*	-3.43	-3.60	-1.80
AL01006A1H12	peptide transport protein	4.00E-25	*Arabidopsis thaliana*	-2.06	2.31	1.13
AL01006A2A10	heavy-metal-associated domain-containing protein	3.00E-27	*Arabidopsis thaliana*	2.76	3.74	4.13
AL01006A2E01	patellin 1	2.00E-54	*Cucurbita pepo*	-3.68	-3.86	-3.74
AL01006B1D01	farnesylated protein 3; metal ion binding	3.00E-61	*Arabidopsis thaliana*	-2.02	-2.28	-2.30
AL01006B1D09	coated vesicle membrane protein	5.00E-89	*Triticum aestivum*	2.38	2.48	2.46
AL01006B2F09	chloroplast ATP/ADP translocator	2.00E-94	*Pisum sativum*	2.17	1.97	2.21
AL01006B2G11	translocase inner membrane subunit 44-2	7.00E-22	*Arabidopsis thaliana*	-2.01	-1.42	-1.55
**Cell Division**						
AL010001000G09	FtsZ protein	2.00E-102	*Gentiana lutea*	-2.54	-2.69	-2.24
AL01004X1A02	phagocytosis and cell motility protein ELMO1	8.00E-59	*Oryza sativa*	9.33	4.06	1.81
**Cytoskelaton**						
AL01005A2B04	alpha-tubulin 7	3.00E-18	*Populus tremuloides*	2.78	2.03	1.18
AL01006B2D03	Caltractin (Centrin)	3.00E-24	*Atriplex nummularia*	1.62	1.66	2.35
AL01003X1A01	caltractin / centrin	3.00E-72	*Arabidopsis thaliana*	1.43	1.59	2.02
AL01003X1D03	prolyl 4-hydroxylase alpha subunit-like	2.00E-07	*Musa acuminata*	-1.24	-1.46	-2.62
**Cell Wall and Metabolism**						
AL01.48.C1.Contig45	caffeoyl-CoA O-methyltransferase	4.00E-69	*Populus sieboldii*	4.63	10.50	8.90
AL01006B1C05	copper-containing amine oxidase	5.00E-66	*Solanum lycopersicum*	9.51	6.88	1.64
AL01006B1D10	expansin-like protein	1.00E-34	*Solanum tuberosum*	1.23	1.07	5.75
AL01004X1D12	fiber protein Fb19	2.00E-50	*Gossypium barbadense*	10.11	9.95	12.39
AL01006B1E08	galactosyltransferase	2.00E-05	*Arabidopsis thaliana*	3.17	4.48	4.04
AL01006A2E04	hydroxyproline-rich glycoprotein family protein	2.00E-23	*Arabidopsis thaliana*	2.16	2.04	2.63
AL01005B1B11	invertase/pectin methylesterase inhibitor	3.00E-33	*Arabidopsis thaliana*	-11.64	-12.63	-13.30
AL01006A1H06	LIM domain protein PLIM1	2.00E-65	*Solanum lycopersicum*	2.91	5.98	4.73
AL01004X1B06	microtubule-associated protein	6.00E-48	*Arabidopsis thaliana*	1.85	1.96	2.21
AL01005A2C07	nitrate-induced NOI protein	3.00E-22	*Arabidopsis thaliana*	1.77	2.13	2.32
AL01.34.C1.Contig33	omega-6 fatty acid desaturase	5.00E-53	*Cucurbita pepo*	-1.28	-1.69	-4.33
AL01005B2A05	pEARLI 1 / extensin-like protein	1.00E-23	*Arabidopsis thaliana*	13.86	12.79	11.46
AL01.84.C1.Contig80	pectin methylesterase	2.00E-94	*Nicotiana tabacum*	-1.48	-6.22	-4.61
AL01.58.C1.Contig55	phloem filament protein PP1	9.00E-21	*Cucurbita maxima*	4.68	-3.55	-3.65
AL01006B2D06	plastid Tic40	2.00E-22	*Ricinus communis*	-2.33	-2.23	-2.17
AL01003X1D01	proline-rich protein	1.00E-11	*Glycine max*	-5.10	-14.45	-20.16
AL01005A2E10	ripening-related protein	5.00E-38	*Arabidopsis thaliana*	-2.49	-3.17	-1.12
**DNA, RNA Related and Gene Expression**						
AL010002000C11	29 kDa ribonucleoprotein A, chloroplast precursor	5.00E-68	*Nicotiana sylvestris*	-2.49	-2.60	-1.46
AL01005A1A10	Auxin-responsive protein IAA22	1.00E-05	*Oryza sativa*	6.53	4.16	2.60
AL01005B2C10	bZIP transcription factor	2.00E-19	*Nicotiana tabacum*	1.94	2.06	1.42
AL01005A2E09	Ca(2+)-dependent DNase	3.00E-102	*Populus tomentosa*	1.94	3.26	3.35
AL01005A1E10	CCAAT-box binding factor HAP5 homolog	6.00E-34	*Daucus carota*	2.05	2.18	2.09
AL010001000C02	EREBP-4 like protein	7.00E-50	*Arabidopsis thaliana*	-1.61	-2.74	-2.77
AL01006B2H09	ERF-like protein	2.00E-63	*Cucumis melo*	3.46	2.35	3.01
AL01005A1B06	ethylene insensitive (EIN3/EIL)-like transcription regulator	8.00E-29	*Fagus sylvatica*	-2.90	-2.84	-3.20
AL01.21.C1.Contig21	jasmonic acid 2; NAM protein	1.00E-32	*Solanum lycopersicum*	-2.23	-1.86	-1.52
AL01005A2B07	mads-box transcription factor	9.00E-14	*Momordica charantia*	12.13	19.40	21.82
AL01005A2H08	Mini zinc finger 2	5.00E-06	*Arabidopsis thaliana*	14.84	11.29	14.17
AL01006B1A07	MYB transcription factor MYB81	5.00E-27	*Glycine max*	2.38	3.06	1.37

AL01.82.C1.Contig78	NAM (no apical meristem)-like protein	1.00E-26	*Prunus persica*	14.46	20.03	22.43
AL01.70.C1.Contig67	NAM, no apical meristem,-like protein	1.00E-11	*Arabidopsis thaliana*	1.10	1.44	3.06
AL01004X1E11	nam-like protein 14	1.00E-36	*Petunia x hybrida*	4.46	3.43	3.28
AL01006A1D01	PHD finger transcription factor	5.00E-14	*Arabidopsis thaliana*	-1.44	-2.28	-3.84
AL01006A2A08	pre-mRNA processing protein PRP39	5.00E-17	*Oryza sativa*	-2.16	-2.52	-2.97
AL01006B1C11	Ran-binding protein, putative	2.00E-61	*Arabidopsis thaliana*	-2.06	-1.19	1.24
AL01005B1F04	REX1 DNA Repair family protein	1.00E-26	*Oryza sativa*	2.22	2.30	2.21
AL01.29.C1.Contig28	S-RNase	1.00E-23	*Prunus dulcis*	5.40	11.73	9.09
AL010001000D08	tetratricopeptide-repeat thioredoxin-like	2.00E-06	*Arabidopsis thaliana*	-2.67	-2.84	-2.52
AL010001000A04	THUMP domain-containing protein	6.00E-71	*Arabidopsis thaliana*	1.80	2.27	1.85
AL01006A1E06	transcription factor bZIP38	4.00E-66	*Glycine max*	2.03	2.76	2.46
AL01004X1D07	YGL010w-like protein	6.00E-40	*Picea mariana*	2.44	1.41	1.45
AL01005B1E06	zinc finger protein	2.00E-21	*Arabidopsis thaliana*	-10.01	-11.93	-12.76
AL01005B1H11	Zinc finger, CCCH-type	4.00E-35	*Medicago truncatula*	2.21	1.55	1.44
**Signal Transduction**						
AL010001000C07	abscisic acid induced protein	1.00E-39	*Arabidopsis thaliana*	2.14	1.93	1.46
AL01.37.C1.Contig36	abscisic acid response protein	1.00E-11	*Cucumis melo*	13.28	11.75	11.13
AL01004X1H01	ACT domain-containing protein	1.00E-63	*Arabidopsis thaliana*	-1.43	-1.89	-2.16
AL01003X1F11	Arabidopsis Rab GTPase homolog C1); GTP binding	2.00E-12	*Arabidopsis thaliana*	-1.66	-2.09	-2.18
AL010001000D09	auxin-repressed protein-like protein ARP1	2.00E-35	*Manihot esculenta*	2.98	1.19	-1.89
AL01.59.C1.Contig56	Avr9/Cf-9 rapidly elicited protein 146	9.00E-13	*Nicotiana tabacum*	4.19	3.85	4.38
AL01.30.C1.Contig29	BURP domain containing protein	2.00E-53	*Arabidopsis thaliana*	30.78	13.26	1.56
AL01006B2D05	calcium binding atopy-related autoantigen 1	5.00E-10	*Oryza sativa*	1.16	-2.20	-2.32
AL01005A1C09	calcium-binding EF-hand family protein	3.00E-36	*Oryza sativa*	-6.51	-8.54	-6.97
AL010002000D03	calmodulin	2.00E-24	*Aegiceras corniculatum*	-2.16	-3.19	-2.00
AL01006A1F11	calmodulin-binding protein	1.00E-75	*Arabidopsis thaliana*	1.91	2.10	2.36
AL01006A2G09	CBL-interacting protein kinase 1	1.00E-05	*Populus trichocarpa*	6.47	29.80	6.42
AL01.28.C1.Contig27	CBL-interacting protein kinase 1	3.00E-66	*Populus trichocarpa*	4.56	19.61	4.18
AL010001000B12	cis-zeatin O-glucosyltransferase	3.00E-08	*Sorghum bicolor*	12.73	7.16	2.84
AL01.17.C1.Contig17	copper chaperone	1.00E-24	*Populus alba*	4.31	2.99	2.22
AL01005B1A12	copper chaperone	3.00E-41	*Oryza sativa*	-1.58	1.32	2.45
AL010002000A06	copper-binding family protein	4.00E-21	*Arabidopsis thaliana*	-2.16	-2.69	-4.03
AL010001000G04	cyclin dependent kinase A	8.00E-96	*Camellia sinensis*	-1.37	-1.77	-2.04
AL01006A2A03	defense; calmodulin binding	2.00E-68	*Arabidopsis thaliana*	-1.61	-1.96	-3.01
AL01006A1H04	elicitor-inducible protein EIG-J7	1.00E-55	*Capsicum annuum*	-1.31	-3.01	-2.46
AL01006B1H12	enzyme-forming ethylene (ACC oxidase)	1.00E-54	*Cucumis melo*	6.36	3.32	9.21
AL01006A2H11	ethylene receptor; Cm-ETR1	4.00E-95	*Cucumis melo*	2.11	1.78	1.54
AL01.20.C1.Contig20	eukaryotic translation initiation factor 5A	4.00E-87	*Rosa chinensis*	-2.02	-1.99	-2.12
AL01006B1B06	IMPAIRED IN BABA-INDUCED STERILITY 1;kinase	1.00E-29	*Arabidopsis thaliana*	-1.96	-2.11	-1.93
AL01006B1E12	leucine-rich receptor-like protein kinase	4.00E-59	*Arabidopsis thaliana*	1.70	-1.28	-3.01
AL01005A1H02	leucine-rich repeat transmembrane protein kinase	2.00E-34	*Arabidopsis thaliana*	-3.71	-4.43	-5.07
AL01.25.C1.Contig25	MFT-like protein	4.00E-16	*Vitis vinifera*	1.94	2.99	1.69
AL01006A2F12	MILDEW RESISTANCE LOCUS O 1;calmodulin binding	2.00E-126	*Arabidopsis thaliana*	1.67	3.16	2.44
AL01005A2A06	OXIDATIVE SIGNAL-INDUCIBLE1; kinase	7.00E-56	*Arabidopsis thaliana*	-2.19	-1.18	1.23
AL01006B1H11	PERK1-like protein kinase	3.00E-81	*Nicotiana tabacum*	-3.68	-3.11	-2.35
AL01006A2D02	phototropic-responsive NPH3 family protein	6.00E-37	*Arabidopsis thaliana*	-4.81	-3.06	-8.68
AL01004X1H04	phytoalexin-deficient 4-1 protein	5.00E-58	*Solanum tuberosum*	-2.02	-2.24	-3.24
AL01.46.C1.Contig44	Pi starvation-induced protein	4.00E-34	*Cicer arietinum*	3.27	4.21	2.94
AL01003X1F07	protein kinase	2.00E-25	*Medicago truncatula*	-7.62	-8.51	-8.33
AL01006B1C07	protein kinase family protein	7.00E-66	*Arabidopsis thaliana*	-2.21	-3.32	-3.64
AL01005A2D01	putative Avr9/Cf-9 rapidly elicited protein	8.00E-55	*Oryza sativa*	-2.28	-2.33	-2.50
AL01006B1A05	putative His-Asp phosphotransfer protein	3.00E-27	*Pisum sativum*	-1.50	-2.02	-2.61
AL010002000C07	putative Ras-related GTP-binding protein	2.00E-63	*Cucumis sativus*	2.03	1.22	1.28
AL01003X1A03	Response regulator receiver	3.00E-05	*Crocosphaera watsonii*	-5.59	-5.13	-5.18
AL01005A1D08	serine/threonine protein phosphatase 2A	1.00E-10	*Arabidopsis thaliana*	-1.55	-2.38	-2.46
AL010002000C05	transducin family protein	6.00E-45	*Arabidopsis thaliana*	1.83	2.21	1.85
AL01006A1G04	transducin family protein / WD-40 repeat family protein	2.00E-37	*Arabidopsis thaliana*	1.76	13.13	8.27
AL01006A2E05	WD repeat domain 48	2.00E-06	*Ostreococcus tauri*	2.22	2.36	1.70
AL01005A2A11	WD-40 repeat protein-like protein	7.00E-64	*Solanum tuberosum*	1.99	2.11	2.03
**Defense and Stress Related Proteins**						
AL01.87.C1.Contig83	Citrus Tristeza Virus Resistance Gene	2.00E-09	*Poncirus trifoliata*	3.70	-1.76	-2.15
AL01.44.C1.Contig42	dessication-related protein	2.00E-111	*Medicago truncatula*	1.59	18.99	15.32
AL01005B1A06	DnaJ protein	1.00E-22	*Camellia sinensis*	-2.53	-2.27	-3.76
AL01.0.C1.Contig1	early nodulin 93	8.00E-26	*Glycine max*	2.33	1.65	1.13

AL01006A1D11	harpin-induced family protein	6.00E-51	*Arabidopsis thaliana*	-1.75	-4.28	-4.15
AL01006B2G07	harpin-induced protein 1	7.00E-06	*Oryza sativa*	1.29	1.27	2.74
AL01006B2E11	heat shock family protein	5.00E-09	*Arabidopsis thaliana*	-5.14	-4.71	-3.94
AL01006B1E05	nitrate responsive NOI protein	7.00E-15	*Arabidopsis thaliana*	2.17	2.27	2.74
AL01.69.C1.Contig66	nodulin family protein	2.00E-39	*Arabidopsis thaliana*	8.13	14.48	11.64
AL01005A2B11	nodulin MtN21	8.00E-05	*Oryza sativa*	2.14	2.37	-1.60
AL01003X1E03	nodulin MtN21 family protein	3.00E-05	*Arabidopsis thaliana*	12.12	19.00	14.87
AL01.33.C1.Contig32	pathogenesis-related protein	7.00E-55	*Eutrema wasabi*	-1.53	2.73	1.48
AL010001000A06	PMR5 (POWDERY MILDEW RESISTANT 5)	6.00E-34	*Arabidopsis thaliana*	-2.28	-4.93	-6.25
AL01005B1D09	putative DnaJ protein	2.00E-30	*Camellia sinensis*	-2.49	-2.72	-3.03
AL01.4.C1.Contig5	PVR3-like protein	1.00E-12	*Ananas comosus*	-3.82	-12.59	-2.14
AL010001000F05	seed maturation protein PM39	1.00E-14	*Glycine max*	1.86	4.05	2.18
AL01005A1F10	silverleaf whitefly-induced protein	7.00E-41	*Cucurbita pepo*	41.04	20.24	20.38
AL01003X1B01	small molecular heat shock protein 10	7.00E-38	*Nelumbo nucifera*	4.34	2.99	2.98
AL010002000A03	snakin-1	2.00E-16	*Solanum tuberosum*	-1.49	-3.04	-1.93
AL01006A1C09	universal stress / early nodulin family protein	4.00E-58	*Arabidopsis thaliana*	15.09	10.60	10.77
AL01006A2D05	universal stress / early nodulin family protein	6.00E-23	*Arabidopsis thaliana*	9.61	6.55	6.98
AL01006B2E09	wound induced protein-like	4.00E-07	*Vitis vinifera*	2.15	1.25	2.24
**Secondary Metabolism**						
AL01005B1D07	flavanone 3-hydroxylase	1.00E-18	*Saussurea medusa*	16.24	26.40	25.09
AL01004X1C05	glutathione S-transferase	4.00E-60	*Glycine max*	2.27	4.48	7.37
AL01005A2B03	glutathione S-transferase	1.00E-12	*Pisum sativum*	1.78	3.34	5.00
AL01005B2G03	glutathione S-transferase	5.00E-18	*Cucurbita maxima*	-2.35	1.61	3.09
AL01005A2F12	phytoene desaturase	1.00E-56	*Momordica charantia*	-2.62	-2.11	-1.69
AL01006B1H02	phytoene synthase, chloroplast precursor (MEL5)	2.00E-60	*Cucumis melo*	-7.97	-3.58	-3.30
AL01004X1H07	relA/spoT homologous protein RSH2	1.00E-17	*Oryza sativa*	-6.81	-5.05	-4.85
**Postranslational Modification**						
AL01005B2A11	NAD+ ADP-ribosyltransferase	7.00E-29	*Arabidopsis thaliana*	2.91	4.19	4.38
**Function Unclear**						
AL01003X1D08	dormant bud-associated protein Drm1	2.00E-05	*Pisum sativum*	2.43	2.22	1.91
AL01004X1C01	DOWNSTREAM TARGET OF AGL15 2	1.00E-77	*Arabidopsis thaliana*	-1.12	-1.83	-2.21
AL010001000F04	embryonic 1 beta-globin	1.00E-28	*Monodelphis domestica*	3.01	2.31	2.99
AL01004X1H02	NLI interacting factor (NIF) family protein	3.00E-39	*Vitis vinifera*	1.33	2.80	3.08
AL010002000A11	pentatricopeptide (PPR) repeat-containing protein	7.00E-11	*Arabidopsis thaliana*	-1.64	-2.27	-1.72
AL01005B1A09	PLASTID TRANSCRIPTIONALLY ACTIVE17	8.00E-91	*Arabidopsis thaliana*	-2.35	-2.61	-1.23
**No significant homology or Unknown function**						
AL010002000G05	Unknown function	NA	NA	-2.67	-3.24	-2.92
AL01004X1F10	Unknown function	NA	NA	-4.33	-22.88	-2.24
AL01005B1G08	Unknown function	NA	NA	-4.03	-3.29	-3.99
AL01005B1H02	Unknown function	NA	NA	-2.25	-1.51	-1.66
AL01006B2B02	Unknown function	NA	NA	-3.22	-3.42	-3.28
AL01003X1F10	Unknown function	NA	NA	-2.01	-2.15	-1.93
AL01005B2H12	Unknown function	NA	NA	-1.94	-1.98	-3.06
AL01003X1G05	Unknown function	NA	NA	-2.95	-6.07	-6.61
AL01005A2A04	Unknown function	NA	NA	-2.15	-3.15	-6.66
AL01003X1F05	Unknown function	NA	NA	-2.28	-2.99	-3.54
AL01004X1A05	Unknown function	NA	NA	-1.38	-2.29	-3.07
AL01004X1B12	Unknown function	NA	NA	-1.82	-2.56	-2.67
AL01005B2H07	Unknown function	NA	NA	1.58	-2.28	-2.09
AL01006B2G12	Unknown function	NA	NA	-1.70	-1.77	-2.12
AL01006A2E12	Unknown function	NA	NA	2.28	-1.51	-2.79
AL01.18.C1.Contig18	Unknown function	NA	NA	5.74	5.26	4.88
AL01005A2C11	Unknown function	NA	NA	2.15	2.06	1.14
AL01005B2G09	Unknown function	NA	NA	3.26	1.82	-1.25
AL01006A2C07	Unknown function	NA	NA	4.25	3.80	4.02
AL01006A1B10	Unknown function	NA	NA	2.10	1.42	1.47
AL01.67.C1.Contig64	Unknown function	NA	NA	4.29	1.67	4.74
AL01003X1B06	Unknown function	NA	NA	6.17	8.59	7.06
AL01003X1H06	Unknown function	NA	NA	2.21	2.44	2.88
AL01006B1B12	Unknown function	NA	NA	2.37	2.03	2.46
AL01006B2G01	Unknown function	NA	NA	2.39	2.06	3.25
AL01.43.C1.Contig41	Unknown function	NA	NA	6.22	8.91	6.80

AL01005A1G10	Unknown function	NA	NA	1.36	3.34	3.66
AL010002000B09	Unknown function	NA	NA	1.08	2.33	7.90
AL010002000B12	No significant homology	NA	NA	-2.32	-2.44	-2.85
AL010002000H01	No significant homology	NA	NA	-2.50	-1.44	-1.26
AL01003X1B11	No significant homology	NA	NA	-3.82	-3.65	-3.23
AL01003X1C07	No significant homology	NA	NA	-2.41	-1.32	-1.27
AL01003X1C11	No significant homology	NA	NA	-2.06	-1.46	-1.52
AL01003X1D09	No significant homology	NA	NA	-2.75	-2.53	-2.48
AL01003X1E10	No significant homology	NA	NA	-2.06	-2.26	-2.08
AL01003X1E11	No significant homology	NA	NA	-2.15	-2.61	-2.31
AL010001000B02	No significant homology	NA	NA	-2.59	-1.62	-1.44
AL01004X1C04	No significant homology	NA	NA	-2.14	-1.81	-1.97
AL01004X1C10	No significant homology	NA	NA	-2.37	-1.58	-1.36
AL01004X1F02	No significant homology	NA	NA	-2.64	-2.64	-2.09
AL01004X1F05	No significant homology	NA	NA	-2.12	-2.17	-2.11
AL01005B1E09	No significant homology	NA	NA	-2.25	-2.35	-2.47
AL01005B2G06	No significant homology	NA	NA	-2.49	-3.00	-2.72
AL01006A1B02	No significant homology	NA	NA	-3.15	-2.21	-2.56
AL01006A1D08	No significant homology	NA	NA	-2.43	-1.53	-1.37
AL01006A1G11	No significant homology	NA	NA	-2.54	-1.98	-1.98
AL01006A2A09	No significant homology	NA	NA	-3.59	-3.48	-3.52
AL01006A2C02	No significant homology	NA	NA	-2.03	-1.84	-1.70
AL01006A2G01	No significant homology	NA	NA	-2.01	-2.30	-2.16
AL01006B2C09	No significant homology	NA	NA	-2.03	-1.47	-1.29
AL01006B2F07	No significant homology	NA	NA	-2.48	-1.41	-2.03
AL01006B2G04	No significant homology	NA	NA	-2.07	-1.40	-1.26
AL01006B2H01	No significant homology	NA	NA	-2.30	1.04	1.27
AL01006B2H06	No significant homology	NA	NA	-2.76	-2.00	-1.63
AL01005A2D11	No significant homology	NA	NA	-3.71	-3.26	-1.92
AL010001000B11	No significant homology	NA	NA	-6.24	-5.92	-9.75
AL01004X1F11	No significant homology	NA	NA	-4.10	-3.65	-2.31
AL01005A2B02	No significant homology	NA	NA	-4.38	-5.37	-4.60
AL01005B2B08	No significant homology	NA	NA	-7.12	-9.03	-10.06
AL01006A1A10	No significant homology	NA	NA	-8.89	-8.24	-5.94
AL01006A1D12	No significant homology	NA	NA	-21.46	-6.35	1.16
AL01006A1F10	No significant homology	NA	NA	-10.29	-5.85	-5.58
AL01006A2B11	No significant homology	NA	NA	-6.02	-3.73	-2.62
AL01003X1B12	No significant homology	NA	NA	-2.01	-1.75	-1.52
AL01006B2C03	No significant homology	NA	NA	-2.17	-2.20	-2.26
AL01005A1H07	No significant homology	NA	NA	-2.2381	-2.6413	-2.3111
AL01005A2B01	No significant homology	NA	NA	-2.2878	-2.9455	-2.3941
AL01006B1B04	No significant homology	NA	NA	-11.93	-18.35	-21.46
AL01005B2D06	No significant homology	NA	NA	-2.78	-3.78	-3.38
AL010002000G02	No significant homology	NA	NA	-1.53	-2.43	-2.30
AL01004X1D03	No significant homology	NA	NA	-1.24	-2.29	-3.35
AL01004X1F03	No significant homology	NA	NA	-2.58	-4.31	-5.54
AL01005B1B10	No significant homology	NA	NA	-1.66	-1.91	-2.32
AL01006A2C10	No significant homology	NA	NA	-1.63	-2.64	-2.35
AL01006A2C12	No significant homology	NA	NA	1.32	-2.46	-2.56
AL01006B1B01	No significant homology	NA	NA	-1.79	-2.73	-3.15
AL01.60.C1.Contig57	No significant homology	NA	NA	-8.51	-11.79	-31.45
AL01005A1E09	No significant homology	NA	NA	-1.43	-4.43	-7.22
AL01003X1B08	No significant homology	NA	NA	1.20	-2.31	-2.40
AL01003X1G08	No significant homology	NA	NA	-1.90	-2.40	-2.48
AL01003X1H07	No significant homology	NA	NA	-1.29	-1.67	-2.06
AL01004X1B02	No significant homology	NA	NA	-2.78	-7.05	-6.62
AL010002000A02	No significant homology	NA	NA	-2.10	-2.71	-3.38
AL010002000H09	No significant homology	NA	NA	-2.57	-8.08	-4.21
AL01005A1G12	No significant homology	NA	NA	-1.7538	-2.1915	-2.1137
AL01005B1E05	No significant homology	NA	NA	1.56	-10.00	-9.69
AL01006A1E05	No significant homology	NA	NA	2.61	1.20	1.97
AL01.24.C1.Contig24	No significant homology	NA	NA	4.13	3.23	2.83
AL01.3.C1.Contig3	No significant homology	NA	NA	12.53	14.01	10.35
AL01.3.C2.Contig4	No significant homology	NA	NA	15.12	16.36	12.61
AL01.42.C1.Contig40	No significant homology	NA	NA	2.34	2.05	1.36
AL01.54.C1.Contig51	No significant homology	NA	NA	5.21	4.36	3.19

AL010001000C05	No significant homology	NA	NA	4.77	1.62	2.36
AL010002000E03	No significant homology	NA	NA	4.83	1.15	1.19
AL010002000G10	No significant homology	NA	NA	5.79	3.82	3.11
AL01003X1A05	No significant homology	NA	NA	9.76	1.69	-1.70
AL01005A1C02	No significant homology	NA	NA	3.40	3.28	2.16
AL01005A2E03	No significant homology	NA	NA	2.08	1.59	1.61
AL01005A2G11	No significant homology	NA	NA	3.98	1.56	1.65
AL01005B1G01	No significant homology	NA	NA	6.46	3.14	6.31
AL01005B1G09	No significant homology	NA	NA	3.29	2.43	1.54
AL01006B2C02	No significant homology	NA	NA	19.22	20.95	15.81
AL01006B1C01	No significant homology	NA	NA	2.45	1.22	1.32
AL01005A1E03	No significant homology	NA	NA	1.83	2.24	1.83
AL01.31.C1.Contig30	No significant homology	NA	NA	4.93	4.47	5.81
AL01.32.C1.Contig31	No significant homology	NA	NA	2.22	2.52	2.55
AL01003X1D07	No significant homology	NA	NA	2.29	2.42	2.53
AL01003X1E07	No significant homology	NA	NA	6.31	6.44	7.23
AL01005A1B09	No significant homology	NA	NA	3.04	3.20	3.61
AL01005A1E02	No significant homology	NA	NA	4.26	4.68	5.54
AL01005A1G03	No significant homology	NA	NA	1.65	1.94	2.03
AL01005A2E06	No significant homology	NA	NA	4.46	3.20	5.30
AL01006B1H09	No significant homology	NA	NA	2.20	1.51	4.56
AL01006B2F11	No significant homology	NA	NA	3.22	3.93	3.35
AL01005B2D01	No significant homology	NA	NA	2.30	3.09	4.38
AL01006A2C03	No significant homology	NA	NA	2.94	5.67	4.65
AL010001000E07	No significant homology	NA	NA	-1.40	3.65	1.25
AL01005A1H01	No significant homology	NA	NA	1.44	4.94	3.82
AL01005A2C12	No significant homology	NA	NA	-1.46	-1.20	2.62
AL01005B1C11	No significant homology	NA	NA	-2.91	1.23	1.72
AL01006A1F01	No significant homology	NA	NA	1.79	5.03	15.47
AL01006B1A02	No significant homology	NA	NA	1.20	1.42	2.23
AL01006B1F02	No significant homology	NA	NA	1.03	2.30	1.86
AL01005B2H09	No significant homology	NA	NA	1.30	1.42	2.85
