# Supplementary material for: Gene expression in developing watermelon fruit
Source: BMC Genomics. 2008 Jun 5;9:275. doi: 10.1186/1471-2164-9-275 (PMC2440768; doi:10.1186/1471-2164-9-275)
Supplement: Additional file 2 — Differential modulation of 127 2x-ESTs was confirmed by Q-PCR using two additional biological replicates. One hundred twenty-seven 2x-ESTs that show differential modulation in the microarray (Biorep 1) were confirmed by Q-PCR (Biorep 2 & Biorep 3). 2x-ESTs induced at least two-fold were highlighted in red whereas 2x-ESTs repressed at least two-fold were highlighted in green. Q-PCR fold inductions are shown for each 2x-EST for green, pink, and red flesh as compared to leaf. [file 1471-2164-9-275-S2.pdf]

Accesion Number	EST-unigene	Biorep1 green Microarray	Biorep1 pink Microarray	Biorep1 red Microarray	Biorep 2 green Q-PCR	Biorep 2 pink Q-PCR	Biorep 2 red Q-PCR	Biorep 3 green Q-PCR	Biorep 3 pink Q-PCR	Biorep 3 red Q-PCR
**Primary Metabolism**										
AL01.68.C1.Contig65	3-oxo-5-alpha-steroid 4-dehydrogenase family	10.64	14.83	10.28	4011.71	3114.96	1351.18	337.79	2320.15	8779.97
AL01004X1H09	brassinosteroid-6-oxidase	4.64	2.32	1.98	-1.18	3.33	1.37	-1.02	8	11.51
AL01005A1G02	chloroplast ribosome recycling factor protein	-3.30	-3.23	-3.19	-12.42	-9.75	-12.55	-7.36	-16.91	-27.00
AL01005B2B06	chloroplast small heat shock protein	8.82	9.43	10.04	9.25	5.33	1.00	-1.39	60.34	62.90
AL010001000F09	copine-related	3.13	3.03	3.55	12.25	8.57	2.71	12.17	6.77	11.08
AL01005B1E08	cytochrome P450 protein	12.51	16.29	5.39	19.16	33.94	37.92	159.79	535.60	36.00
AL01.73.C1.Contig70	cytochrome P450-like protein	6.92	7.89	4.67	39.26	89.57	82.42	66.49	59.51	43.87
AL01005B1B12	disulfide bond formation protein	1.42	4.34	4.09	8.40	8.88	3.00	4.29	4.87	14.12
AL010002000C03	ent-kaurenoic acid oxidase	2.87	2.51	2.04	-1.52	3.67	2.06	9.06	9.61	8.22
AL01005B2C07	ent-kaurene synthase	-2.03	-1.38	-1.50	-3.56	1.31	-1.78	1.33	1.12	1.51
AL01.57.C1.Contig54	epicotyl-specific tissue protein	7.88	1.36	-1.20	-1.38	54.19	897.64	25409.51	1282.73	1247.65
AL01.88.C1.Contig84	lactoylglutathione lyase family protein	9.05	8.80	4.22	15.67	30.48	16.11	12.34	14.88	9.71
AL01006A2G06	malate dehydrogenase	2.42	1.98	-1.24	13.22	33.24	16.45	28.74	19.16	8.03
AL01006B2E04	peroxidase ATP2a	3.84	2.86	-2.65	38.19	310.83	533.74	709.18	196.04	85.04
AL01005B2H10	plastid ribosomal protein L11	-5.17	-5.25	-4.27	-21.93	-14.67	-24.93	-12.68	-19.16	-14.62
AL01.9.C1.Contig9	pyruvate decarboxylase	20.45	25.44	23.66	297.14	546.85	75.06	47.67	408.73	1541.37
AL01.63.C1.Contig60	specific tissue protein 2	25.74	5.68	3.15	5.06	544.96	2998.45	12503.12	3565.78	2583.30
**Amino Acid Synthesis, Processing**										
AL01004X1A03	60S ribosomal protein L37a	2.04	1.43	1.79	-4.03	1.22	-1.67	-1.39	-1.44	-1.89
AL01004X1F08	alanine:glyoxylate aminotransferase 2	3.83	1.76	-1.23	1.12	2.23	1.36	55.72	3.13	1.85
AL01005B2E08	cysteine protease 1	2.92	1.60	1.33	1.84	8.2	8.6	20.25	24.25	11.04
AL01.7.C1.Contig7	hECT ubiquitin-protein ligase 3	1.05	-1.89	-3.92	-5.64	1.34	-1.33	34.9	-2.99	1.65
AL01005A2G12	OTU-like cysteine protease family protein	-2.35	-1.71	-1.51	-5.45	-1.11	-2.32	-1.24	1.58	2.35
AL010002000E11	S-adenosylmethionine decarboxylase	-4.02	-1.39	-1.46	-7.81	-1.01	1.27	1.07	-1.05	2.10
AL01005A1H10	subtilisin	9.54	3.03	-1.14	39.40	99.04	29.65			
AL01.74.C1.Contig71	subtilisin-type protease precursor	21.75	20.23	7.99	102.54	243.03	236.39	617.37	484.38	308.69
AL01006A1G08	ubiquitin (UBA) domain-containing protein	2.77	3.77	4.34	10.06	12.95	3.77	4.91	13.32	35.14
AL010002000D06	ubiquitin-conjugating enzyme 8	2.32	2.35	1.99	3.75	7.31	2.66	4.42	6.73	6.17
AL01005A1D01	ubiquitin-protein ligase	2.88	3.67	3.39	3.32	14.37	7.86	6.82	20.25	46.85
AL01006B2B08	ubiquitin-protein ligase 7	1.04	2.23	1.71	-1.96	2.62	1.89	3.39	6.13	10.67
**Membrane and Transport**										
AL01.83.C1.Contig79	annexin	2.11	3.93	3.97	14.22	19.43	8.11	14.27	13.55	30.80
AL01006B1D09	coated vesicle membrane protein	2.38	2.48	2.46	-3.68	1.21	1.01	1.8	-1.11	1.26
AL01003X1C05	copine-related	2.97	2.65	3.50	9.65	4.52	1.73	19.49	6.73	19.36
AL01005A2F02	Embryo-specific 3;lipoxygenase	2.84	7.84	6.88	6.99	22.94	2.94	2.84	18.90	30.38
AL01.50.C1.Contig47	globulin-like protein	25.70	41.54	12.26	3578.15	5025.32	891.44	8.28	2530.14	7777.01
AL01006A2A10	heavy-metal-associated protein	2.76	3.74	4.13	12.13	14.47	6.50	2.52	6.43	24.17
AL01005A1D04	membrane protein	1.12	4.20	4.65	3.54	3.69	-3.97	-4.35	2.05	5.62
AL01006A1H12	peptide transport protein	-2.06	2.31	1.13	18.32	26.08	9.09	-3.10	74.03	18.32
AL01005B2C12	plasma membrane H+-ATPase	7.68	10.81	12.85	52.71	73.52	23.51	1389.16	1108.97	4938.99
AL01006A1B03	putative indole-3-glycerol phosphate synthase	-2.73	-2.26	-2.45	-9.42	-3.36	-7.34	-4.07	-3.69	1.4
AL01006A1B09	sugar transporter	6.39	6.96	1.44	39.26	146.02	115.36	130.24	118.19	52.89
AL01005B2A04	sugar transporter-like protein	-6.88	-6.62	-5.85	-25.9	-5.64	-7.65	-9.48	-7.97	-6.7
AL01003X1B03	tonoplast monosaccharide transporter	3.02	2.80	2.09	-1.73	4.53	5.37	6.04	13.41	2.78
AL010002000F03	type IIB calcium ATPase	-3.93	-2.24	-2.99	-18.70	-2.61	-2.41	-1.65	-2.87	-15.94
**Cytoskelaton**										
AL01005A2B04	alpha-tubulin 7	2.78	2.03	1.18	1.13	1.78	-1.13	-1.36	2.38	2.63
**Cell Wall and Metabolism**										
AL01.48.C1.Contig45	caffeoyl-CoA O-methyltransferase	4.63	10.50	8.90	1.39	10.67	6.43	1.18	4.74	39.40
AL01006B1C05	copper-containing amine oxidase	9.51	6.88	1.64	6.43	69.07	24.76	6.02	126.24	46.37
AL01006B1D10	expansin-like protein	1.23	1.07	5.75	22.78	43.26	6.82	17.33	39.12	69.55
AL01004X1D12	fiber protein Fb19	10.11	9.95	12.39	56.10	63.34	27.76	47.34	107.26	112.60
AL01006A2E04	hydroxyproline glycoprotein family protein	2.16	2.04	2.63	8.22	22.86	9.35	41.64	105.05	87.73
AL01005B1B11	invertase/pectin methylesterase inhibitor	-11.64	-12.63	-13.30	-17.63	-1.09	1.97	-1.14	1.14	-9.82
AL01006A1H06	LIM domain protein PLIM1	2.91	5.98	4.73	8.31	12.08	5.92	6.13	13.45	44.48
AL01004X1B06	microtubule-associated protein	1.85	1.96	2.21	1.88	7.36	3.58	11.16	9.38	14.27
AL01005B2A05	pEARLI 1 / extensin-like protein	13.86	12.79	11.46	390.72	1024.00	831.75	7332.05	8335.19	28231.14
AL01.84.C1.Contig80	pectin methylesterase	-1.48	-6.22	-4.61	-1038.29	-3.32	-2.58	3.06	-15.83	-903.89
AL01.58.C1.Contig55	phloem filament protein PP1	4.68	-3.55	-3.65	-32.33	1.14	5.15	522.76	6.94	2.39
AL01003X1D01	proline-rich protein	-5.10	-14.45	-20.16	-148.57	-1.81	3.39	13.41	-2.03	-38.45
**DNA, RNA Related, Gene Expression**										
AL01005A1A10	Auxin-responsive protein IAA22	6.53	4.16	2.60	54.76	79.62	99.04	1.27	184.82	96.67
AL01005A1E10	CCAAT-box binding factor HAP5 homolog	2.05	2.18	2.09	1.57	7.14	3.06	2.54	5.33	11.31

AL010001000C02	EREBP-4 like protein	-1.61	-2.74	-2.77	-8.31	-1.18	-1.29	1.82	1.27	-1.13
AL01006B2H09	ERF-like protein	3.46	2.35	3.01	118.19	99.39	80.17	318.47	188.71	224.41
AL01005A1B06	ethylene insensitive transcription regulator	-2.90	-2.84	-3.20	-8.85	1.01	-2.28	1.66	-1.18	1.04
AL01.21.C1.Contig21	jasmonic acid 2; NAM protein	-2.23	-1.86	-1.52	5.62	35.38	2.12	4.61	12.21	30.7
AL01005A2B07	mads-box transcription factor	12.13	19.40	21.82	319.57	310.83	143.01	324.03	1160.07	3743.05
AL01005A2H08	mini zinc finger 2	14.84	11.29	14.17	282.09	272.48	132.97	133.44	349.71	1140.14
AL01006B1A07	MYB transcription factor MYB81	2.38	3.06	1.37	1.89	9.32	5.68	15.67	12.91	2.22
AL01.82.C1.Contig78	NAM (no apical meristem)-like protein	14.46	20.03	22.43	13.36	15.14	4.56	30.91	517.35	1045.52
AL01.70.C1.Contig67	NAM, no apical meristem,-like protein	1.10	1.44	3.06	3.39	11.35	5.6	14.72	22.94	136.71
AL01004X1E11	nam-like protein 14	4.46	3.43	3.28	4.48	34.3	3.41	1704.34	968.76	820.3
AL01.29.C1.Contig28	S-RNase	5.40	11.73	9.09	135.30	1168.14	566.13	33.47	3756.05	7967.99
AL010001000D08	tetratricopeptide thioredoxin	-2.67	-2.84	-2.52	-204.36	-5.68	-3.01	-1.41	-154.34	-70.52
AL01006A1E06	transcription factor bZIP38	2.03	2.76	2.46	-1.67	3.04	2.12	1.42	3.94	2.76
AL01004X1D07	YGL010w-like protein	2.44	1.41	1.45	2.20	3.81	2.27	29.55	5.19	5.26
AL01005B1E06	zinc finger protein	-10.01	-11.93	-12.76	-44.17	-42.08	-48.00	-131.14	-116.97	-139.10

Signal Transduction

AL01.37.C1.Contig36	abscisic acid response protein	13.28	11.75	11.13	118.60	263.20	203.66	33.01	757.45	1924.14
AL010001000D09	auxin-repressed protein-like protein ARP1	2.98	1.19	-1.89	24.76	43.11	29.65	100.43	29.86	2.92
AL01.30.C1.Contig29	BURP domain containing protein	30.78	13.26	1.56	6.92	66.72	144.51	173.65	42.22	16.00
AL01006B2D05	calcium binding atopy-related autoantigen 1	1.16	-2.20	-2.32	-6.02	-1.34	-1.11	5.6	2	-1.41
AL01005A1C09	calcium-binding EF-hand family protein	-6.51	-8.54	-6.97	-458.25	-49.35	-33.47	-2.69	-64.45	-155.96
AL01006A2A03	calmodulin binding	-1.61	-1.96	-3.01	-8.31	-1.01	-1.32	3.39	-1.54	-1.94
AL01006A2G09	CBL-interacting protein kinase 1	6.47	29.80	6.42	85.92	233.13	100.43	1.61	441.11	2574.36
AL01.28.C1.Contig27	CBL-interacting protein kinase 1	4.56	19.61	4.18	342.51	1618.00	489.44	-3.90	219.03	1217.75
AL010001000B12	cis-zeatin O-glucosyltransferase	12.73	7.16	2.84	52.71	349.71	504.95	5367.37	3704.34	2377.12
AL01006B1H12	enzyme-forming ethylene (ACC oxidase)	6.36	3.32	9.21	15.35	2.36	1.43	116.57	2.83	65.57
AL01006A2H11	ethylene receptor; Cm-ETR1	2.11	1.78	1.54	-1.24	4.53	3.85	12.77	5.15	11.71
AL01006A2F12	mildew resistance;calmodulin binding	1.67	3.16	2.44	-1.49	4.32	1.16	3.08	4.42	2.8
AL01.46.C1.Contig44	Pi starvation-induced protein	3.27	4.21	2.94	50.04	118.60	45.10	31.34	104.69	210.84
AL01006A1G04	WD-40 repeat family protein	1.76	13.13	8.27	8.91	32.79	7.73	-1.03	43.41	114.96

Defense / Stress Proteins

AL01.87.C1.Contig83	Citrus Tristeza Virus Resistance Gene	3.70	-1.76	-2.15	-1.27	7.36	4.82	5.72	-2.11	-17.33
AL01.44.C1.Contig42	dessication-related protein	1.59	18.99	15.32	79.34	29.24	-4.44	-1.08	778.74	6539.66
AL01005B1A06	DnaJ protein	-2.53	-2.27	-3.76	-1.68	2.24	-1.02	2.81	1.49	1.85
AL01006A1D11	harpin-induced family protein	-1.75	-4.28	-4.15	-15.95	-2.78	-2.68	1.95	-2.24	-12.86
AL01006B2E11	heat shock family protein	-5.14	-4.71	-3.94	-1.63	-1.65	-2.74	3.68	-1.19	1.00
AL01.69.C1.Contig66	nodulin family protein	8.13	14.48	11.64	2352.53	4167.60	823.14	2.06	2172.29	3315.48
AL01003X1E03	nodulin MtN21 family protein	12.12	19.00	14.87				14.32	552.56	1247.65
AL01.33.C1.Contig32	pathogenesis-related protein	-1.53	2.73	1.48	8.78	23.26	4.77	-30.80	125.80	51.27
AL010001000A06	PMR5 (POWDERY MILDEW RESISTANT)	-2.28	-4.93	-6.25	-71.26	-3.69	-3.56	-1.3	-4.63	-6.82
AL01005B1D09	putative DnaJ protein	-2.49	-2.72	-3.03	-3.16	2.62	1.19	4.81	3.99	2.11
AL01.4.C1.Contig5	PVR3-like protein	-3.82	-12.59	-2.14	-3.82	-4.76	-1.85	10.41	-7.14	-4.59
AL01005A1F10	silverleaf whitefly-induced protein	41.04	20.24	20.38	184.82	381.36	721.57	2217.93	179.15	160.90
AL01006A1C09	universal stress / early nodulin family protein	15.09	10.60	10.77				58.08	100.43	205.79
AL01006A2D05	universal stress / early nodulin family protein	9.61	6.55	6.98	5.86	14.93	5.17	89.57	44.94	13.41

Secondary Metabolism

AL01005B1D07	flavanone 3-hydroxylase	16.24	26.40	25.09	388.02	262.29	15.08			
AL01004X1C05	glutathione S-transferase	2.27	4.48	7.37	74.80	34.42	6.89	1.40	6.11	5.46
AL01005A2B03	glutathione S-transferase	1.78	3.34	5.00	1.19	2.24	1.03	-1.29	4.64	23.67
AL01005B2G03	glutathione S-transferase	-2.35	1.61	3.09	8.25	3.43	-2.11	-2.82	2.85	6.21
AL01005A2F12	phytoene desaturase	-2.62	-2.11	-1.69	-16.85	-4.32	-4.71	-3.19	-4.42	-1.24
AL01006B1H02	phytoene synthase, chloroplast precursor	-7.97	-3.58	-3.30	-5.31	-3.00	-13.45	-21.33	-3.15	3.24

No significant homology or Unknown function

AL01006A2E12	Unknown function	2.28	-1.51	-2.79	5.76	11.79	6.84	183.55	139.58	9.99
AL01.67.C1.Contig64	Unknown function	4.29	1.67	4.74	5.88	2.89	19.49	20.32	27.19	11.47
AL01003X1B06	Unknown function	6.17	8.59	7.06	25.99	31.12	7.31	23.83	48.34	220.56
AL01.43.C1.Contig41	Unknown function	6.22	8.91	6.80	20.18	28.54	6.68	-2.71	21.56	52.71
AL01006B1B04	No significant homology	-11.93	-18.35	-21.46	-14.83	-6.84	-31.45	-1.20	-8.78	-7.44
AL01.60.C1.Contig57	No significant homology	-8.51	-11.79	-31.45	-26.45	1.14	11.75	17.33	-2.54	-31.56
AL01005B1E05	No significant homology	1.56	-10.00	-9.69	-265.95	-21.78	-11.20	7.57	-36.50	-57.48
AL01.3.C1.Contig3	No significant homology	12.53	14.01	10.35	5202.54	40763.8	27650.16	82.71	3565.78	4688.79
AL01.3.C2.Contig4	No significant homology	15.12	16.36	12.61	3848.29	30257.39	21693.87	84.74	3553.44	4344.58
AL01.54.C1.Contig51	No significant homology	5.21	4.36	3.19	1.84	7.84	7.86	15.24	5.78	7.84
AL010002000G10	No significant homology	5.79	3.82	3.11	1.93	4.13	3.26	13.45	6.54	3.04
AL01003X1A05	No significant homology	9.76	1.69	-1.70	1.42	31.78	54.76	168.90	79.89	1.06
AL01005A1C02	No significant homology	3.40	3.28	2.16	10.52	13.98	8.40	7.89	6.00	16.28
AL01006B2C02	No significant homology	19.22	20.95	15.81	4389.98	33456.53	23010.42	430.54	21769.19	34041.34
AL01003X1E07	No significant homology	6.31	6.44	7.23	14.22	24.50	12.30	8.28	49.52	105.42
AL01006B2F11	No significant homology	3.22	3.93	3.35	3.19	5.01	2.45	5.39	8.57	53.63
AL01005B2D01	No significant homology	2.30	3.09	4.38	3.38	9.61	4.66	3.1	14.03	4.99

AL01006A2C03	No significant homology	2.94	5.67	4.65	9.78	6.25	1.03	1.05	32.79	98.02
AL010001000E07	No significant homology	-1.40	3.65	1.25	68.36	48.84	5.48	-1.48	77.44	27.28
AL01006B1A02	No significant homology	1.20	1.42	2.23	2.98	3.42	2.08	6.25	2.93	8.40
