# Supplementary material for: Gene expression in developing watermelon fruit
Source: BMC Genomics. 2008 Jun 5;9:275. doi: 10.1186/1471-2164-9-275 (PMC2440768; doi:10.1186/1471-2164-9-275)
Supplement: Additional file 3 — 2x-ESTs that are differentially modulated at a specific fruit stage compared to leaf as determined by microarray analysis. One hundred and seventy-six 2x-ESTs that exhibit induction in one or more fruit types with a false discovery rate (FDR) of less than 0.05 were identified. [file 1471-2164-9-275-S3.pdf]

Accesion Number	EST-unigene	Microarray	FDR	Microarray	FDR	Microarray	FDR	Timepoint
**Primary Metabolism**								
AL01.41.C1.Contig39	formate dehydrogenase	2.04	1.54E-12	2.74	1.62E-14	2.25	2.98E-13	12, 24 &36
AL01.61.C1.Contig58	lipase class 3 family protein	12.26	3.30E-16	7.95	3.65E-15	4.84	1.32E-13	12, 24 &36
AL01.63.C1.Contig60	specific tissue protein 2	25.74	8.44E-19	5.68	1.46E-15	3.15	3.37E-13	12, 24 &36
AL01.68.C1.Contig65	3-oxo-5-alpha-steroid 4-dehydrogenase family	10.64	1.08E-17	14.83	2.13E-18	10.28	1.70E-17	12, 24 &36
AL01.73.C1.Contig70	cytochrome P450-like protein	6.92	1.04E-16	7.89	4.71E-17	4.67	1.96E-15	12, 24 &36
AL01.88.C1.Contig84	lactoylglutathione lyase family protein	9.05	5.03E-17	8.80	6.10E-17	4.22	1.17E-14	12, 24 &36
AL01.9.C1.Contig9	pyruvate decarboxylase	20.45	3.41E-20	25.44	2.35E-20	23.66	4.23E-20	12, 24 &36
AL010001000C11	ferredoxin III	3.14	1.79E-15	2.90	4.57E-15	3.83	2.38E-16	12, 24 &36
AL010001000F09	copine-related	3.13	7.32E-14	3.03	1.03E-13	3.55	1.70E-14	12, 24 &36
AL010002000A05	cytochrome c oxidase	2.67	5.04E-13	2.53	1.02E-12	3.02	9.77E-14	12, 24 &36
AL010002000C03	ent-kaurenoic acid oxidase	2.87	2.33E-12	2.51	1.37E-11	2.04	4.08E-10	12, 24 &36
AL01005B1E08	cytochrome P450 protein	12.51	1.63E-18	16.29	3.31E-19	5.39	2.17E-16	12, 24 &36
AL01005B2B03	short-chain dehydrogenase/reductase 2	12.32	2.34E-18	4.72	9.16E-16	8.67	1.70E-17	12, 24 &36
AL01005B2B06	chloroplast small heat shock protein	8.82	3.67E-18	9.43	2.18E-18	10.04	2.00E-18	12, 24 &36
AL01005B2F12	Glutamine synthetase cytosolic isozyme 1	5.06	1.04E-15	3.07	1.28E-13	2.69	6.59E-13	12, 24 &36
AL01004X1H09	brassinosteroid-6-oxidase	4.64	3.37E-16	2.32	8.50E-13	1.98	1.45E-11	12 & 24
AL01006A2B05	urate oxidase	2.61	5.41E-14	2.77	2.35E-14	1.51	3.44E-09	12 & 24
AL01006B2E04	peroxidase ATP2a	3.84	5.00E-15	2.86	1.28E-13	-2.65	3.37E-13	12 & 24
AL01005B1B12	disulfide bond formation protein	1.42	1.08E-07	4.34	7.67E-16	4.09	1.42E-15	12 & 36
AL01.57.C1.Contig54	epicotyl-specific tissue protein	7.88	1.63E-18	1.36	6.42E-08	-1.20	2.72E-05	12 only
AL01006A2G06	malate dehydrogenase	2.42	7.11E-14	1.98	2.14E-12	-1.24	6.62E-06	12 only
AL010001000G11	dihydrolipoamide succinyltransferase	1.20	4.27E-05	1.71	8.27E-11	2.25	3.37E-13	36 only
AL01005A1F08	2-oxo acid dehydrogenase, lipoyl-binding site	1.98	1.44E-12	1.87	4.70E-12	2.50	2.96E-14	36 only
AL01005A2H02	UDP-glucose 6-dehydrogenase	1.32	3.66E-07	1.26	3.99E-06	2.04	1.72E-12	36 only
**Amino Acid Synthesis, Processing, and Protein Degradation**								
AL01.49.C1.Contig46	papain-like cysteine peptidase XBCP3	2.57	1.25E-14	3.74	1.56E-16	3.03	1.54E-15	12, 24 &36
AL01.74.C1.Contig71	subtilisin-type protease precursor	21.75	4.71E-18	20.23	6.03E-18	7.99	6.77E-16	12, 24 &36
AL01003X1E05	serine-type peptidase	2.10	4.42E-12	2.59	1.53E-13	3.25	8.98E-15	12, 24 &36
AL01005A1D01	ubiquitin-protein ligase	2.88	3.04E-14	3.67	1.92E-15	3.39	4.36E-15	12, 24 &36
AL01006A1G08	ubiquitin (UBA)/TS-N domain-containing protein	2.77	2.81E-14	3.77	8.03E-16	4.34	2.38E-16	12, 24 &36
AL010002000D06	ubiquitin-conjugating enzyme 8	2.32	1.37E-13	2.35	1.07E-13	1.99	1.80E-12	12 & 24
AL01005A1H10	subtilisin	9.54	4.31E-18	3.03	3.83E-14	-1.14	0.0026	12 & 24
AL01004X1A03	60S ribosomal protein L37a	2.04	6.28E-12	1.43	4.83E-08	1.79	8.61E-11	12 only
AL01004X1F08	alanine:glyoxylate aminotransferase 2	3.83	2.83E-14	1.76	2.62E-09	-1.23	0.0003524	12 only
AL01005B2E08	cysteine protease 1	2.92	2.59E-15	1.60	1.54E-10	1.33	7.61E-08	12 only
AL01006B2B08	ubiquitin-protein ligase 7	1.04	0.356	2.23	1.31E-11	1.71	2.36E-09	24 only
**Membrane and Transport**								
AL01.50.C1.Contig47	globulin-like protein	25.70	9.38E-20	41.54	2.35E-20	12.26	1.36E-18	12, 24 &36
AL01.55.C1.Contig52	integral membrane family protein	3.27	1.56E-15	2.93	5.46E-15	3.03	3.74E-15	12, 24 &36
AL01.83.C1.Contig79	annexin	2.11	7.50E-13	3.93	2.48E-16	3.97	2.38E-16	12, 24 &36
AL01003X1B03	tonoplast monosaccharide transporter	3.02	7.96E-15	2.80	1.98E-14	2.09	1.67E-12	12, 24 &36
AL01003X1C05	copine-related	2.97	5.78E-14	2.65	2.34E-13	3.50	8.13E-15	12, 24 &36
AL01005A2F02	Embryo-specific 3;lipoxygenase	2.84	5.88E-14	7.84	8.65E-18	6.88	2.31E-17	12, 24 &36
AL01005B2C12	plasma membrane H+-ATPase	7.68	2.34E-18	10.81	3.03E-19	12.85	2.18E-19	12, 24 &36
AL01006A2A10	heavy-metal-associated protein	2.76	3.94E-15	3.74	1.36E-16	4.13	5.68E-17	12, 24 &36
AL01006B1D09	coated vesicle membrane protein	2.38	4.11E-13	2.48	2.17E-13	2.46	2.41E-13	12, 24 &36
AL01006A1B09	sugar transporter	6.39	7.51E-15	6.96	3.97E-15	1.44	8.70E-06	12 & 24
AL01.85.C1.Contig81	GAMMA-SOLUBLE ATTACHMENT PROTEIN	1.47	3.36E-09	2.29	1.31E-13	2.51	3.22E-14	12 & 36
AL01005A1D04	membrane protein	1.12	0.002	4.20	7.56E-17	4.65	3.29E-17	12 & 36
AL01006B2F09	chloroplast ATP/ADP translocator	2.17	7.50E-13	1.97	4.54E-12	2.21	5.45E-13	12 & 36
AL01006A1H12	peptide transport protein	-2.06	7.84E-13	2.31	1.15E-13	1.13	0.0010	24 only
**Cell Division**								
AL01004X1A02	phagocytosis and cell motility protein ELMO1	9.33	2.01E-16	4.06	8.28E-14	1.81	6.69E-09	12 & 24
**Cytoskelaton**								
AL01005A2B04	alpha-tubulin 7	2.78	2.63E-13	2.03	3.35E-11	1.18	0.0007	12 & 24
AL01003X1A01	caltractin / centrin	1.43	8.55E-09	1.59	3.15E-10	2.02	1.05E-12	36 only
AL01006B2D03	Caltractin (Centrin)	1.62	6.19E-11	1.66	3.21E-11	2.35	3.09E-14	36 only
**Cell Wall and Metabolism**								
AL01.48.C1.Contig45	caffeoyl-CoA O-methyltransferase	4.63	8.68E-17	10.50	3.44E-19	8.90	1.14E-18	12, 24 &36
AL01004X1D12	fiber protein Fb19	10.11	1.75E-18	9.95	1.42E-18	12.39	7.24E-19	12, 24 &36
AL01005B2A05	pEARLI 1 / extensin-like protein	13.86	9.77E-20	12.79	1.04E-19	11.46	2.40E-19	12, 24 &36
AL01006A1H06	LIM domain protein PLIM1	2.91	6.61E-13	5.98	7.19E-16	4.73	4.44E-15	12, 24 &36
AL01006A2E04	hydroxyproline-rich glycoprotein protein	2.16	1.01E-12	2.04	2.75E-12	2.63	4.80E-14	12, 24 &36
AL01006B1E08	galactosyltransferase	3.17	3.00E-16	4.48	1.03E-17	4.04	2.71E-17	12, 24 &36
AL01006B1C05	copper-containing amine oxidase	9.51	8.71E-18	6.88	6.66E-17	1.64	3.38E-09	12 & 24
AL01005A2C07	nitrate-induced NOI protein	1.77	2.59E-11	2.13	5.89E-13	2.32	1.49E-13	12 & 36

AL01.58.C1.Contig55	phloem filament protein PP1	4.68	3.28E-12	-3.55	4.16E-11	-3.65	3.19E-11	12 only
AL01004X1B06	microtubule-associated protein	1.85	7.00E-12	1.96	1.86E-12	2.21	2.18E-13	36 only
AL01006B1D10	expansin-like protein	1.23	1.67E-05	1.07	0.0741	5.75	2.34E-17	36 only
**DNA, RNA Related and Gene Expression**								
AL01.29.C1.Contig28	S-RNase	5.40	7.99E-18	11.73	9.31E-20	9.09	4.00E-19	12, 24 &36
AL01.82.C1.Contig78	NAM (no apical meristem)-like protein	14.46	6.28E-18	20.03	1.42E-18	22.43	1.14E-18	12, 24 &36
AL01004X1E11	nam-like protein 14	4.46	1.27E-14	3.43	1.53E-13	3.28	2.58E-13	12, 24 &36
AL01005A1A10	Auxin-responsive protein IAA22	6.53	7.99E-18	4.16	2.53E-16	2.60	4.80E-14	12, 24 &36
AL01005A1E10	CCAAT-box binding factor HAP5 homolog	2.05	1.36E-11	2.18	4.64E-12	2.09	9.33E-12	12, 24 &36
AL01005A2B07	mads-box transcription factor	12.13	3.02E-19	19.40	3.24E-20	21.82	4.23E-20	12, 24 &36
AL01005A2H08	MINI ZINC FINGER 2	14.84	1.82E-17	11.29	7.20E-17	14.17	2.53E-17	12, 24 &36
AL01005B1F04	REX1 DNA Repair family protein	2.22	5.84E-13	2.30	3.00E-13	2.21	5.97E-13	12, 24 &36
AL01006A1E06	transcription factor bZIP38	2.03	7.62E-11	2.76	5.67E-13	2.46	2.87E-12	12, 24 &36
AL01006B2H09	ERF-like protein	3.46	4.94E-15	2.35	6.48E-13	3.01	2.23E-14	12, 24 &36
AL01006B1A07	MYB transcription factor MYB81	2.38	9.70E-13	3.06	3.34E-14	1.37	5.08E-07	12 & 24
AL01005A2E09	Ca(2+)-dependent DNase	1.94	7.54E-12	3.26	3.44E-15	3.35	2.65E-15	12 & 36
AL01004X1D07	YGL010w-like protein	2.44	1.70E-12	1.41	3.54E-07	1.45	1.49E-07	12 only
AL01005B1H11	Zinc finger, CCCH-type	2.21	3.14E-13	1.55	8.76E-10	1.44	9.30E-09	12 only
AL010001000A04	THUMP domain-containing protein	1.80	2.77E-10	2.27	3.18E-12	1.85	1.51E-10	24 only
AL01005B2C10	bZIP transcription factor	1.94	3.51E-11	2.06	1.13E-11	1.42	1.46E-07	24 only
AL01.70.C1.Contig67	NAM, no apical meristem,-like protein	1.10	0.065	1.44	1.55E-06	3.06	8.77E-13	36 only
**Signal Transduction**								
AL01.17.C1.Contig17	copper chaperone	4.31	3.60E-16	2.99	1.54E-14	2.22	9.75E-13	12, 24 &36
AL01.28.C1.Contig27	CBL-interacting protein kinase 1	4.56	5.00E-15	19.61	8.75E-19	4.18	1.03E-14	12, 24 &36
AL01.37.C1.Contig36	abscisic acid response protein	13.28	2.75E-18	11.75	4.59E-18	11.13	7.52E-18	12, 24 &36
AL01.46.C1.Contig44	Pi starvation-induced protein	3.27	1.94E-15	4.21	1.56E-16	2.94	6.35E-15	12, 24 &36
AL01.59.C1.Contig56	Avr9/Cf-9 rapidly elicited protein 146	4.19	4.59E-16	3.85	9.81E-16	4.38	3.04E-16	12, 24 &36
AL010001000B12	cis-zeatin O-glucosyltransferase	12.73	7.74E-20	7.16	7.87E-19	2.84	2.69E-15	12, 24 &36
AL01006A2G09	CBL-interacting protein kinase 1	6.47	3.91E-13	29.80	1.48E-16	6.42	3.82E-13	12, 24 &36
AL01006B1H12	enzyme-forming ethylene (ACC oxidase)	6.36	8.73E-17	3.32	2.35E-14	9.21	1.06E-17	12, 24 &36
AL01.30.C1.Contig29	BURP domain containing protein	30.78	7.50E-21	13.26	9.40E-20	1.56	6.67E-10	12 & 24
AL01006A2E05	WD repeat domain 48	2.22	1.16E-13	2.36	4.15E-14	1.70	2.51E-11	12 & 24
AL01005A2A11	WD-40 repeat protein-like protein	1.99	9.40E-12	2.11	3.06E-12	2.03	6.50E-12	12 & 36
AL01006A1F11	calmodulin-binding protein	1.91	1.57E-11	2.10	2.52E-12	2.36	3.43E-13	12 & 36
AL01006A1G04	transducin family protein	1.76	2.86E-07	13.13	6.10E-16	8.27	8.13E-15	12 & 36
AL01006A2F12	MILDEW RESISTANCE calmodulin binding	1.67	4.89E-09	3.16	1.10E-13	2.44	3.31E-12	12 & 36
AL010001000C07	abscisic acid induced protein	2.14	7.81E-13	1.93	5.47E-12	1.46	7.04E-09	12 only
AL010001000D09	auxin-repressed protein-like protein ARP1	2.98	6.04E-16	1.19	1.13E-05	-1.89	7.45E-13	12 only
AL010002000C07	putative Ras-related GTP-binding protein	2.03	2.50E-11	1.22	0.0001	1.28	1.26E-05	12 only
AL01006A2H11	ethylene receptor; Cm-ETR1	2.11	5.03E-12	1.78	1.60E-10	1.54	7.04E-09	12 only
AL01.25.C1.Contig25	MFT-like protein	1.94	4.96E-10	2.99	5.87E-13	1.69	9.60E-09	24 only
AL010002000C05	transducin family protein	1.83	1.02E-11	2.21	2.66E-13	1.85	8.47E-12	24 only
AL01005B1A12	copper chaperone	-1.58	1.37E-06	1.32	0.0004	2.45	3.63E-10	36 only
**Defense and Stress Related Proteins**								
AL01.69.C1.Contig66	nodulin family protein	8.13	1.09E-16	14.48	4.82E-18	11.64	1.78E-17	12, 24 &36
AL01003X1B01	small molecular heat shock protein 10	4.34	3.39E-17	2.99	1.45E-15	2.98	1.61E-15	12, 24 &36
AL01003X1E03	nodulin MtN21 family protein	12.12	5.09E-19	19.00	5.64E-20	14.87	2.18E-19	12, 24 &36
AL01005A1F10	silverleaf whitefly-induced protein	41.04	5.00E-21	20.24	2.97E-20	20.38	4.23E-20	12, 24 &36
AL01006A1C09	universal stress / early nodulin family protein	15.09	6.17E-18	10.60	3.71E-17	10.77	3.35E-17	12, 24 &36
AL01006A2D05	universal stress / early nodulin family protein	9.61	2.75E-18	6.55	2.73E-17	6.98	2.05E-17	12, 24 &36
AL01006B1E05	nitrate responsive NOI protein	2.17	1.76E-12	2.27	7.82E-13	2.74	5.06E-14	12, 24 &36
AL01005A2B11	nodulin MtN21	2.14	2.89E-10	2.37	4.67E-11	-1.60	1.22E-07	12 & 24
AL01.44.C1.Contig42	dessication-related protein	1.59	8.55E-09	18.99	3.03E-19	15.32	9.67E-19	12 & 36
AL010001000F05	seed maturation protein PM39	1.86	1.10E-10	4.05	2.35E-15	2.18	5.28E-12	12 & 36
AL01006B2E09	wound induced protein-like	2.15	2.97E-10	1.25	0.0004	2.24	1.46E-10	12 & 36
AL01.0.C1.Contig1	early nodulin 93	2.33	4.15E-12	1.65	4.27E-09	1.13	0.0065	12 only
AL01.87.C1.Contig83	Citrus Tristeza Virus Resistance Gene	3.70	7.15E-14	-1.76	4.85E-09	-2.15	9.46E-11	12 only
AL01.33.C1.Contig32	pathogenesis-related protein	-1.53	1.17E-08	2.73	1.33E-13	1.48	3.24E-08	24 only
AL01006B2G07	harpin-induced protein 1	1.29	0.000	1.27	0.0002	2.74	6.67E-12	36 only
**Secondary Metabolism**								
AL01004X1C05	glutathione S-transferase	2.27	2.41E-13	4.48	8.61E-17	7.37	2.87E-18	12, 24 &36
AL01005B1D07	flavanone 3-hydroxylase	16.24	3.39E-17	26.40	4.57E-18	25.09	6.66E-18	12, 24 &36
AL01005A2B03	glutathione S-transferase	1.78	4.20E-09	3.34	2.53E-13	5.00	5.66E-15	12 & 36
AL01005B2G03	glutathione S-transferase	-2.35	5.72E-13	1.61	1.37E-09	3.09	1.35E-14	36 only
**Postranslational Modification**								
AL01005B2A11	NAD+ ADP-ribosyltransferase	2.91	5.00E-15	4.19	1.13E-16	4.38	7.96E-17	12, 24 &36
**Function Unclear**								
AL010001000F04	embryonic 1 beta-globin	3.01	2.18E-14	2.31	8.11E-13	2.99	2.24E-14	12, 24 &36

AL01003X1D08	dormant bud-associated protein Drm1	2.43	8.81E-13	2.22	3.61E-12	1.91	6.32E-11	12 & 24
AL01004X1H02	NLI interacting factor (NIF) family protein	1.33	1.24E-06	2.80	7.99E-14	3.08	2.41E-14	12 & 36
**No significant homology or Unknown function**								
AL01.18.C1.Contig18	Unknown function	5.74	1.83E-17	5.26	3.75E-17	4.88	6.77E-17	12, 24 &36
AL01.24.C1.Contig24	No significant homology	4.13	1.40E-12	3.23	1.73E-11	2.83	8.61E-11	12, 24 &36
AL01.3.C1.Contig3	No significant homology	12.53	3.41E-20	14.01	2.35E-20	10.35	9.39E-20	12, 24 &36
AL01.3.C2.Contig4	No significant homology	15.12	5.09E-19	16.36	2.37E-19	12.61	9.67E-19	12, 24 &36
AL01.31.C1.Contig30	No significant homology	4.93	1.62E-14	4.47	3.56E-14	5.81	4.35E-15	12, 24 &36
AL01.32.C1.Contig31	No significant homology	2.22	1.16E-10	2.52	1.61E-11	2.55	1.42E-11	12, 24 &36
AL01.43.C1.Contig41	Unknown function	6.22	4.71E-18	8.91	4.50E-19	6.80	3.13E-18	12, 24 &36
AL01.54.C1.Contig51	No significant homology	5.21	6.06E-17	4.36	2.48E-16	3.19	5.56E-15	12, 24 &36
AL010002000G10	No significant homology	5.79	1.12E-16	3.82	3.65E-15	3.11	3.09E-14	12, 24 &36
AL01003X1B06	Unknown function	6.17	2.03E-18	8.59	2.32E-19	7.06	9.67E-19	12, 24 &36
AL01003X1D07	No significant homology	2.29	4.65E-10	2.42	1.86E-10	2.53	9.64E-11	12, 24 &36
AL01003X1E07	No significant homology	6.31	6.10E-16	6.44	5.27E-16	7.23	2.50E-16	12, 24 &36
AL01003X1H06	Unknown function	2.21	1.86E-13	2.44	3.62E-14	2.88	3.85E-15	12, 24 &36
AL01005A1B09	No significant homology	3.04	8.82E-12	3.20	4.64E-12	3.61	1.23E-12	12, 24 &36
AL01005A1C02	No significant homology	3.40	1.22E-14	3.28	1.70E-14	2.16	5.51E-12	12, 24 &36
AL01005A1E02	No significant homology	4.26	3.60E-16	4.68	1.56E-16	5.54	4.54E-17	12, 24 &36
AL01005A2E06	No significant homology	4.46	3.17E-16	3.20	8.03E-15	5.30	7.96E-17	12, 24 &36
AL01005B1G01	No significant homology	6.46	2.34E-17	3.14	1.31E-14	6.31	2.91E-17	12, 24 &36
AL01005B2D01	No significant homology	2.30	5.84E-13	3.09	1.06E-14	4.38	3.04E-16	12, 24 &36
AL01006A2C03	No significant homology	2.94	5.86E-13	5.67	1.01E-15	4.65	5.07E-15	12, 24 &36
AL01006A2C07	Unknown function	4.25	1.05E-16	3.80	2.82E-16	4.02	1.96E-16	12, 24 &36
AL01006B1B12	Unknown function	2.37	2.66E-12	2.03	3.56E-11	2.46	1.44E-12	12, 24 &36
AL01006B2C02	No significant homology	19.22	9.77E-20	20.95	5.64E-20	15.81	2.18E-19	12, 24 &36
AL01006B2F11	No significant homology	3.22	9.49E-14	3.93	1.17E-14	3.35	5.86E-14	12, 24 &36
AL01006B2G01	Unknown function	2.39	3.11E-14	2.06	3.43E-13	3.25	5.76E-16	12, 24 &36
AL01.42.C1.Contig40	No significant homology	2.34	1.80E-11	2.05	1.66E-10	1.36	6.47E-06	12 & 24
AL01005A2C11	Unknown function	2.15	1.14E-09	2.06	2.47E-09	1.14	0.0287	12 & 24
AL01005B1G09	No significant homology	3.29	3.11E-14	2.43	1.41E-12	1.54	2.05E-08	12 & 24
AL01.67.C1.Contig64	Unknown function	4.29	1.01E-15	1.67	1.13E-09	4.74	4.32E-16	12 & 36
AL010001000C05	No significant homology	4.77	1.91E-14	1.62	8.59E-08	2.36	5.15E-11	12 & 36
AL010002000B09	Unknown function	1.08	0.069	2.33	6.87E-12	7.90	6.16E-17	12 & 36
AL01005A1G10	Unknown function	1.36	7.95E-07	3.34	1.31E-14	3.66	4.99E-15	12 & 36
AL01005A1H01	No significant homology	1.44	2.00E-06	4.94	9.27E-15	3.82	9.37E-14	12 & 36
AL01006A1F01	No significant homology	1.79	2.13E-07	5.03	2.83E-13	15.47	2.95E-16	12 & 36
AL01006B1H09	No significant homology	2.20	2.78E-12	1.51	1.39E-08	4.56	5.04E-16	12 & 36
AL010002000E03	No significant homology	4.83	3.00E-16	1.15	0.0017	1.19	0.0003	12 only
AL01003X1A05	No significant homology	9.76	8.69E-14	1.69	1.05E-05	-1.70	9.64E-06	12 only
AL01005A2E03	No significant homology	2.08	2.97E-11	1.59	1.12E-08	1.61	9.12E-09	12 only
AL01005A2G11	No significant homology	3.98	2.86E-14	1.56	7.90E-08	1.65	1.93E-08	12 only
AL01005B2G09	Unknown function	3.26	7.73E-11	1.82	4.40E-07	-1.25	0.0074	12 only
AL01006A1B10	Unknown function	2.10	5.44E-09	1.42	3.90E-05	1.47	1.31E-05	12 only
AL01006A1E05	No significant homology	2.61	8.25E-11	1.20	0.0049	1.97	7.59E-09	12 only
AL01006A2E12	Unknown function	2.28	9.64E-12	-1.51	7.35E-08	-2.79	4.78E-13	12 only
AL01006B1C01	No significant homology	2.45	3.10E-13	1.22	4.26E-05	1.32	1.10E-06	12 only
AL010001000E07	No significant homology	-1.40	0.000	3.65	1.44E-11	1.25	0.0041	24 only
AL01005A1E03	No significant homology	1.83	1.88E-10	2.24	3.97E-12	1.83	1.90E-10	24 only
AL01006B1F02	No significant homology	1.03	0.488	2.30	4.30E-11	1.86	2.13E-09	24 only
AL01005A1G03	No significant homology	1.65	3.52E-08	1.94	9.88E-10	2.03	4.16E-10	36 only
AL01005A2C12	No significant homology	-1.46	6.56E-08	-1.20	0.0003	2.62	3.37E-13	36 only
AL01005B2H09	No significant homology	1.30	4.73E-06	1.42	1.32E-07	2.85	7.12E-14	36 only
AL01006B1A02	No significant homology	1.20	0.001	1.42	7.75E-07	2.23	1.91E-11	36 only
