# Supplementary material for: Gene expression in developing watermelon fruit
Source: BMC Genomics. 2008 Jun 5;9:275. doi: 10.1186/1471-2164-9-275 (PMC2440768; doi:10.1186/1471-2164-9-275)
Supplement: Additional file 4 — Primer sets and amplicon size for 2x-ESTs used in Q-PCR. [file 1471-2164-9-275-S4.pdf]

EST ID	FORWARD	REVERSE	AMPLICON
AL01.3.C1.Contig3	AGTAATGGCGGTCGTGCTACTTGT	TCTTGGGACAGCCATCATCTCTGA	133 bp
AL01.3.C2.Contig4	AGTAATGGCGGTCGTGCTACTTGT	TCTTGGGACAGCCATCATCTCTGA	133 bp
AL01.4.C1.Contig5	TGTATGCATGTAGACCGTCGGTGA	AAGAAAGAGCTCCCGAGTTTCGGT	126 bp
AL01.7.C1.Contig7	TGGCAACATTTGCAAGACAGAGGG	AGAGGCCAAACTCCAAAGACGATG	189 bp
AL01.9.C1.Contig9	AACGGCTGTGATTGCTGAAACAGG	TCCAAGTGTAGCACCAACTGACCA	130 bp
AL01.21.C1.Contig21	CTTTGAAATTCCGGCGGACTCTGT	AACGTAGAGATCGGCGATGGTGTT	148 bp
AL01.28.C1.Contig27	GGCATGGCAGTTTCAGTAAGGTCA	TGGCCAACACCTCGTATAGCCTAA	186 bp
AL01.30.C1.Contig29	AGCTTGTGGCTTGTCACACTATGC	TTGAAAGGAAGGATGGGTAGGGCT	179 bp
AL01.33.C1.Contig32	TGCTTCCTTCCTCTCTCGCTCAAA	CCCTTGCGTTCGTTGGCATATTGT	145 bp
AL01.37.C1.Contig36	AGAGAAGAAGCACCACAAGCACCT	AACTTGTGTTCATGGCTGTGCTGG	126 bp
AL01.43.C1.Contig41	AAGGGTTTCTTCCGGACCCAACTT	AGAGGATCCAAGACCCACATGCTT	101 bp
AL01.44.C1.Contig42	AAGACTACAGGGAACGTGCTTGCT	ATTTAGCAATGTGGCCATCGGCTC	151 bp
AL01.46.C1.Contig44	CTACAAGAATGCTGCCAGTGGCTT	AGTGAAGATGGGAAGGCCCTGAAT	137 bp
AL01.48.C1.Contig45	TCGTTGCATTTCAGCTAATGGCCG	TTGGATAGGCGTTGGCTTCCAGTA	99 bp
AL01.50.C1.Contig47	ATATACAACGCCGTTCCCAATGCC	TCGAGAACTGCAGTCAACCCAGAT	110 bp
AL01.54.C1.Contig51	TATGATTGGTGAATCCGAGCGTGG	TCGCTCCACTTTCACTCTCTCAGT	151 bp
AL01.57.C1.Contig54	AGTTTAGAGCTACCGCCAACTGCT	TGCTTTCCTCTACGGCGGTTGTTT	145 bp
AL01.58.C1.Contig55	TGGAAGAAGTTGCAAGGTTTGCGG	GTCCAAAGCAATCCTTGGCCTGAA	153 bp
AL01.60.C1.Contig57	TACCACTACAAATCTCCGCCACCA	CCTCCAGGCTTTCCTGTTCCAAAT	164 bp
AL01.63.C1.Contig60	AGCCAAAGAGCACTCTGCTGATGA	TGCTTCTCCTTCTTTCCTTGGGCT	112 bp
AL01.67.C1.Contig64	GGCTCAAGTGGTTGCTTGTTCGTT	TGGTGGGTGGATGGTTGACTTTGA	156 bp
AL01.68.C1.Contig65	TCAAGCGGGACTTTGAGGTACTGT	TCAATGGATGGCTCTGGAAGTGCT	154 bp
AL01.69.C1.Contig66	TGCTTGCCGGGATCCTCTATGATT	CCCAACCACACATGAACAAGCCAT	122 bp
AL01.70.C1.Contig67	TCCCAAGAAACAGGGCTGACTACT	GCACAGTTGAAGCCGAAGGATTGT	118 bp
AL01.73.C1.Contig70	ACAGAGTTCTCTGCTTCATCGGCT	GATGTGTCTGTTCCTGCACGCAAT	117 bp
AL01.74.C1.Contig71	ACAATCCTGCCCACTGTGACCATA	AGCTTGAATCATTGCCAAGCCAGG	163 bp
AL01.82.C1.Contig78	CTCGCATAGGCCAATGGATCATCA	GGCCACCATTCCTTGAAGCAAACT	160 bp
AL01.83.C1.Contig79	ATGCAATCAAGAGGGTCGGGAAGA	TAATCGCCAGACTCCTTGGACACA	154 bp
AL01.84.C1.Contig80	ACGTCCACTCCAACCGTCAATTCT	GCGTGAATGTCGCAGTCTTGGAAA	106 bp
AL01.87.C1.Contig83	GTGCTTTGCCAGATGCTTTGCTTG	TTGAGACCTTCTGGACAGCAACCT	99 bp
AL01.88.C1.Contig84	GGCTGTTTGGTTACGGCATTGGAA	TACTACCGCCCGCACGTAATCAAT	176 bp
AL01006B2C02	AGTAATGGCGGTCGTGCTACTTGT	TCTTGGGACAGCCATCATCTCTGA	133 bp
AL01003X1B06	GCTAGATGGTAGCGAGATCGTTGT	GCAGCAGCAGTAGCAGCATTCTTT	146 bp
AL01006A2D05	AAGGGAGAAGTTGTGTGAGGCTGT	AATAGCCAACGAGCAAGGAGTGGA	131 bp
AL010001000A06	TGGCCTATTACAAAGGCCTCACCA	TTCCGAGGAAGAAGACGGTGGTTT	87 bp
AL010001000B12	TGCAGAGAAGTTAGGGAATGTCGT	ACCAGTCTAAGGAAAGTTCGCAGG	186 bp
AL010001000C02	TCATGACTATTCGACCAGTGGCGT	GATCAAGGCAGGTTGTGCCAATCA	101 bp
AL010001000C05	CTTGCTGAGTTCATCAAAGCTTGCC	TGACCTAGAGAGAGAGAGAGAGAG	154 bp
AL010001000D08	GGAGATTCCAGCTTCCAAACCACA	GCCCAATGGAGAATCAAGTGGAGT	127 bp
AL010001000D09	TCACCACCAAGCCCTTCATCCTTA	AGACGCTCCTCCAAACATTGTCCT	159 bp
AL010001000E07	AGCCTCGACGGCGTTCTTAAAGTT	ATAGTCAGTCCCATTCTGGAAAGC	87 bp
AL010001000F09	GCTGCAAATTCAAGGGAGGAACCA	TTGCTGTGGCACGTAACTCTGACT	156 bp
AL010002000A03	AAGTGCTTCTTTCATGGACACCGC	AAGGGCAATGGTGCTTGCCATAAC	170 bp
AL010002000G10	AGAGGCCAAAGAGGAAACAGTGGA	TTTGGCTTCTCTTCCTCAGCTGGT	126 bp
AL010002000A02	CGCGGCCGTTCTTTATCGAAGTTT	TGAGTCCCAATATGCCACCTCCAA	116 bp
AL010002000A06	TGAAGAGCCCTGTTTCTGGTTCCT	ATGGACAGTCAACGAGCGAGTCAA	176 bp
AL010002000C03	TCAGGTCACACTATGATGTGGGCA	TCTTCTTGCTCTGCCTTGGCCTTT	83 bp
AL010002000D06	AACAGTGGAGTCCTGCACTAACCA	TGCCTCGTACTTGCTTCTGTCAGT	137 bp
AL010002000E11	TCTGAGGAAGTTGCCGTCCTTGAT	GCACAGGCAGAATAAACGTGCCAT	116 bp

AL010002000F03	TTTGTGAGCATGCCAGTTGCAGTC	TGGGCCAGAAGGGATTGGTTCATA	102 bp
AL01003X1D01	CCGCTTGTCTTTGCACTGCCATTA	ACTGGTAGCCAGTGGGAACAGTTT	117 bp
AL01003X1D09	ACGAAAGTGGAATGCAAAGAAGCA	TCTCTCTATTTCTTCTTGGTGTTG	80 bp
AL01003X1E07	TGGTGGGTGACAGAGATGATGAGA	TCCTTCACCTATCTCCCTGCTTCA	112 bp
AL01003X1A05	AAGATGAGACGACGATGTGTAGCG	CTCACCAGTTGGATTCTTCTCTTG	132 bp
AL01003X1B03	ACTATGGACCATCCCTGCCTTGAT	ATTGATGCATTCACTACGCTGCCC	90 bp
AL01003X1C05	TATGCCCGTTCTGTCGGAGTTCAA	TCGACATCGAGGCATTATCCCTGT	137 bp
AL01004X1A03	GAAGGCTGGCATTGTCGGCAAATA	TGTGCTGGCTGACTTCCATCTTCT	86 bp
AL01004X1B06	TGACAGAATTCCGGTGATCGTGGA	TCCTCTTTCGGACCACGTACACAA	125 bp
AL01004X1C05	TTCTCGTCCACAATGGAAGACCCA	AGGTAGAAGGGAAGGGCCATTGTT	95 bp
AL01004X1D07	GGCCGCTTTGCTTTGTTTCGTTTG	ACAACTGAGCTGCCAGTACAACCT	101 bp
AL01004X1D12	AGCTCATCGTTGTTCACGCCAAAC	ACCTCCAACACAACATCATGCACC	180 bp
AL01004X1E11	ACAACTTCTTCTCCCTCCTCATCC	ATTGAATCGGAGAAGCAGGTCACG	86 bp
AL01004X1F08	AGGGTGTTGGAGGAGCTGTTGAAT	GCCAGTTTGAACCTCATCGGCAAT	107 bp
AL01004X1H09	AGGTGGAACTAGACATTGTCCTGGC	TAATGTGGAACCCACTTGGAGCCT	155 bp
AL01005A1A10	GGAGCTTCAACTTGGCCTTGCTTT	AAGTGTTAGACGAAACGCCGCAGA	160 bp
AL01005A1B06	ATCGGCCTGTTGATGCACAGTTTG	AGCAAGCAAGCAAGCTTATGCTCC	170 bp
AL01005A1C02	CCGTTACAAGGCTCATGTATCTGAC	AGCAACTGATGAAATTCAGACATC	94 bp
AL01005A1C09	TGCTAGCTATGGCTAATGGGATGG	ATTGCAGTTTCTCGCTCAACAGCC	97 bp
AL01005A1D01	CGGTTCAGGCAGTTTGCGATTCAT	GACTGCACAAGCACAATGCACAGA	113 bp
AL01005A1E10	CACGAAAGGTTCCATTCCCGTAGT	GTGGCACCGTACATGTTTGCTTGA	135 bp
AL01005A1F10	ATCGTAGCCATCTCAATGCTCTGC	ATCGATGTAGACGAGACCGAAACG	137 bp
AL01005A1G02	ATTCCGTCAAATGCGGCTCCACTA	ATTCCTCGAAACAGGGAGTTGGGT	119 bp
AL01005A1H01	ACTGTCAAGAATGTGGGCAGTTGA	ACTGTCAAGAATGTGGGCAGTTGA	124 bp
AL01005A2B03	AAGCTAGCTGCATGGATTGAGGAG	TCCCGTCGACGTGTTGGGTAATTT	160 bp
AL01005A2B07	GTGATGTGATTTGCAGCTGTGTGTG	GGTCCATGTGGGCACAAGAATCAT	130 bp
AL01005A2D02	TCGAGAAGCCAGAAGAGGCATGAA	ACTCTGCAGTTTACCCATTTGCGG	101 bp
AL01005A2E10	TGTTGGTCTCGACACCGTCTTGAT	CTGCAACTTTGCCAGCATAACCCA	152 bp
AL01005A2F02	ATCGATGATCCGTCTTCAAGGGCA	TTTCCATCCATCGTATCCGCTCCT	123 bp
AL01005A2F12	GTCTGTTTACAAGACTGTGCCCGA	GCTTTCCCGAAAGAACAGCACCTT	140 bp
AL01005A2G12	AGCTTGCAGACGAGCTCAGAGAAA	TGGCGCATGCGTCTAACATACTGA	112 bp
AL01005A2H08	TGTCGAGAATTCATGGCGAGTGG	TGAGGGTGATGAAGAGCAATCGCA	135 bp
AL01005B1A06	TGCTGAGGTAACCGTTGTTTGGTG	TTCCCATCCCATCCATTCTTCCCA	165 bp
AL01005B1B11	GCGCAGCCTTAACCGATCAAGAAA	TTTGCGCACACATCCACCTTCATC	85 bp
AL01005B1B12	AGTGCACTGATGGATTGTGTCGGA	TGCAGATCCAGACCCAAGTGTTCT	134 bp
AL01005B1D07	AAGCAACGGAACATCGAGTCGTGA	TGATGGAGCGATATTGTGGAGGGT	144 bp
AL01005B1D09	TCCACCGCTTCCTCATATGTCTCA	CGGAAGTAATTGTGGCGTGCAGTT	120 bp
AL01005B1D10	AAAGTTCATCGAGGCGATCGGACA	TCAGCAGCAACAGGATTTGGCTTG	111 bp
AL01005B1E05	AAGCTGGAAGGCATGCAATACTGG	ACACCAGTCACAATACTTGGCGCT	103 bp
AL01005B1E06	TGTGACAAATGGGTTCATGGTGCC	TGGTGCAAGTGATGATGGTAGGGA	145 bp
AL01005B1E08	TGGGAGAAGAAAGTGCCCTGGATT	AGGAACAGGGTTGAGAATGAGAGG	178 bp
AL01005B2A04	CCACTTCGAACTAGAGGGAAAGGA	AAGGGAAAGCAATGCAATGGCACC	153 bp
AL01005B2C07	ACGAGGAAACGGGTTACTGTTCCA	ACATTCCTCATCACACTGCGTCCT	121 bp
AL01005B2C12	TCACTGGTGTGATTCCCTTCGTGT	ATATTCACCCGTGCACACCTCACA	115 bp
AL01005B2D01	TATGCCTATCCCTATCCACCTCCT	AGCAACAACATAGAGCTGCAAGGC	139 bp
AL01005B2E08	CGGCCAATGCAAATTCGTAGCTGA	ACCACCTCAAATGCTACGCTGACT	135 bp
AL01005B2G03	TTGGCAACTTCAGCTTTGAGTCCG	AGGGACTTGGCAACACTCTCCTTT	88 bp
AL01005B2H10	ATAAAGCTTGCTCTGGAGGCTGGA	CCTTGTCAGCTGTCTTGGCATTGT	124 bp
AL01005B2A05	AAGGGACACTCTTAAACTCGGCGT	TAGCCTTAATGGCAGTGCAAAGGC	155 bp
AL01005B2B06	AAGGCCGATCTTCCCGGTATCAAA	ACTGCTCCGCTCAATTCTATGCCA	138 bp

AL01006A1B03	TCTGGGCTGTTCACTCCTGATGAT	TCACAATCGACTCGCCAACCAGAA	82 bp
AL01006A1B09	TGGGAGCTGTTCCTTGGGTGATAA	ATCCAAGCACCCAACCAATTCACC	103 bp
AL01006A1D11	AATTTCCTCCTCACTGTCTCCGCT	TGGTTCGGAGAGTGATCTGTTGGT	112 bp
AL01006A1E06	AGGGCTTCTGATACCAACAAGGCT	TTGGAGTTTACCATCAGCCGCAGT	147 bp
AL01006A1G04	AAACTCGTTGAGAAGGCTTCGCGT	GATGGAATGCCATAGTGGAAGGCA	101 bp
AL01006A1G08	TGCAGAGTTGATGAATGGCATCCG	TTCCATCACTACTCGCTTCACGCT	123 bp
AL01006A1H06	ACATAGCGCACGAACATCGACTCT	GTTGCATTCGCAGCCAGTTCTTCT	151 bp
AL01006A1H12	TGCTTGCTGCCTTGACTGTGATTG	AGAACCAGCCTCTCTCTCTCACTT	194 bp
AL01006A2A03	AACTTCAGTGGCGACGAGCTTCTA	AAGTGTAGCGGAAGTGCTGGGTTA	160 bp
AL01006A2A10	ACGAGAAGACGAAGCAGAAGGCAA	TTTCTTCACAATCGCCACCGCATC	131 bp
AL01006A2C03	TGAAAGGCTCCGGGATCTGCATTA	TTACAGCGAGATGAAGGGATGCCA	129 bp
AL01006A2E04	ACCTCCGATCATCTCCCTCCAAAT	GGCATCGCAATGAACTTCGGAACA	118 bp
AL01006A2E12	CTTGTGCCGAAGCAAGCGAAATCT	CCTCCACTCCAATCAAACTGCCAT	157 bp
AL01006A2F12	TTCATTCTTCTGCTCGCTGTGGGA	AAGTGATCATCGGACGGTTGGACT	125 bp
AL01006A2G06	AACGTCACAATCTGAATGCACCGC	AGGGACAAAGAGCTTCCCGTTGTA	118 bp
AL01006A2G09	GCGAATGGAGGATCTTGTAACAGAT	TGCCACGTGACAAAGACAATTAG	136 bp
AL01006A2H11	AGCTGGCATGGATGGGCTAATACT	ACGTTTCGAATAGAACCCGACGCT	98 bp
AL01006B1A02	CCATAACTGCAGTCCTACCTTTCT	TGGGCTTGAAATCCCTCCTTTGAC	108 bp
AL01006B1A07	TTCCAGCAGAGGAGGACGAAATCA	TTCGTCCATTCAGAAGCCTAGCGA	90 bp
AL01006B1C05	ACCGCCGCTAACTTGTTGGATTTG	AAGTTTGAAGGGTATCCTCGCCGT	154 bp
AL01006B1D09	TGGCACTATGGCGATGATGGAGTT	ACGGAGACTTGTTCGTGAAGCAGA	151 bp
AL01006B1D10	AAAGGACCATTGCAGTTGCGGTTC	ATCAATCCAGGTCTCCAGTCAGCA	101 bp
AL01006B1H02	AGGCAGGACTTTCAGATGAGGACA	ACACCGGCCATCTGCTAGCTTTAT	153 bp
AL01006B1H12	TAGTTTGGGTCCAATGGCAACAGC	AGAGGCAAACACAAAGACAAGCCC	126 bp
AL01006B2B08	GGCACATTAAAGGCGGCTGTTCTT	AAACGTTTCCGTCCAAACAGGGTG	81 bp
AL01006B2D05	TGCAGAGCGATTGGAGGAGTTCTT	GCAAAGCTCTTTGTCCGTTGCTGA	122 bp
AL01006B2E04	CCCTCACTGGAGATCAAGGTGAGATT	CAACCCACAGCCACAACCATCATT	149 bp
AL01006B2E11	ACCCGACATCCAAAGATGGGAAGA	AGCTGGCTTTCTTAGGCATCGTGA	134 bp
AL01006B2F11	GCACAAGTAGTTCTGCTCTGTGAGT	CAGGCTACCAACAACTGCAT	132 bp
AL01006B2H09	ATTCCGGGTATGGAGAATTCCGCT	CACGGCGTTCATGTTCTGACCAAA	146 bp
